# Emesis in Rodents: Present or Absent? A Critical Review of the Evidence and Implications for the Use of Rodents in Biomedical Research

**DOI:** 10.3390/biology15010035

**Published:** 2025-12-25

**Authors:** Gustavo Alcantara De Sousa, Masahiro Nemoto, John A. Rudd, Gareth J. Sanger, Paul L. R. Andrews

**Affiliations:** 1 Faculty of Veterinary Medicine, Universidade Nove de Julho, Memorial Campus, São Paulo 01156-050, Brazil; gustavo.alcantara@uni9.edu.br; 2 Department of Pharmacology, Japanese Red Cross Hokkaido College of Nursing, Kitami 090-0011, Japan; nemoto@rchokkaido-cn.ac.jp; 3 School of Biomedical Sciences, Faculty of Medicine, The Chinese University of Hong Kong, Hong Kong, China; jar@cuhk.edu.hk; 4 Blizard Institute, Faculty of Medicine and Dentistry, Queen Mary University of London, London E1 4NS, UK; g.sanger@qmul.ac.uk; 5 Institute of Medical and Biomedical Education, City St George’s, University of London, Tooting Campus, London SW17 0RE, UK

**Keywords:** digestive tract, emetic, oesophageal sphincter, mouse, nausea, rat, retching, rodent, vomiting

## Abstract

Vomiting, a key defence against accidentally ingested toxins, is widely present in mammals. Notably, rodents (e.g., rats, mice) are generally assumed to be unable to vomit, as commonly reported over the last ~100 years, but they are used extensively for biological and particularly biomedical research. However, three recent mouse papers reported ‘emetic-like’ responses induced by substances that are emetics in humans. Therefore, we critically re-examined the literature underpinning this longstanding view, integrating the recent and largely overlooked historical evidence challenging this consensus. We reappraise the ability of rodents to retch and vomit, concluding that whilst there is some published evidence for retching, the ability to vomit is unproven and for a range of physiological and anatomical reasons (e.g., organisation of the gastroesophageal anti-reflux barrier), it would be problematic. Experimentally testable hypotheses to explain the conflicting findings are proposed, together with criteria that should be fulfilled to define a behaviour as retching or vomiting in rodents. A detailed comparison is made of the physiological and anatomical differences between rodents and species known to vomit. We consider the significance of our conclusions for biomedical research, particularly in relation to the digestive tract and central nervous system, and the wider implications for rodent biology.

## Simple Summary

The ability to vomit is an important component of the body’s defensive systems to limit the effects of toxins ingested with food. However, in a notable exception amongst mammals, it is widely reported that rodents (e.g., rats and mice) and lagomorphs (e.g., rabbits) are unable to vomit. This review addresses the validity of this assumption in the light of both historical and recent evidence of behaviours that are apparently analogous to retching and/or vomiting in selected rodent species. The evidence is critically evaluated based on a detailed examination of anatomical, functional and experimental factors. We conclude that whilst there is some limited published evidence for retching, the ability to vomit is not proven. The reasons for this are discussed and include: (i) anatomical constraints in the region of the gastro-oesophageal junction which would impede the bulk ejection of gastric contents during vomiting (especially semi-solids); (ii) neural control of the diaphragm and lower oesophageal sphincter; (iii) possible differences between species with a well-defined vomiting response and those without, in the brainstem pathways coordinating the activity of the muscles involved in retching and vomiting. These conclusions need to be tested experimentally. The wider implications for rodent biology and evolution are discussed.

## 1. Introduction

Nausea and vomiting are components of the body’s defences against ingested toxins (e.g., plant, animal, bacterial). Nausea reduces the intake of potentially contaminated food, induces a learned aversion to that food and is associated with delayed gastric emptying to confine the toxin to the stomach. Vomiting forcibly ejects the gastric contents to reduce the toxic load [1]. However, despite its adaptive significance, over at least the last 100 years, vomiting has been commonly reported as not occurring in rodents, as exemplified by rats and mice (e.g., [2,3,4]). Rodentia are the most abundant and diversified order of extant placental mammals [5,6], accounting for around 40% of all mammalian species [5,6]. Instead, biomedical research on vomiting primarily uses species from the Carnivora (e.g., dog, cat, ferret) and Insectivora (e.g., house musk shrew) orders [2,3,7,8,9]).

In mammals, vomiting, the forceful expulsion of gastric contents through the mouth, is usually preceded by multiple retches, which are rhythmic contractions of specific thoracic and abdominal muscles with no ejection of the gastric contents [10,11,12,13,14]. Together, retching and vomiting are referred to as ‘emesis’ and these mechanical events should be differentiated from nausea, a self-reported sensation in humans [15]. Retching and vomiting have a characteristic signature of muscle activity (particularly the diaphragm, intercostal and abdominal muscles) [16,17]. Retching is characterised by pulses of decreased intra-thoracic pressure with concomitant increases in intra-abdominal pressure, and a closed mouth and glottis ([18] for review). During a vomit, there is a synchronous increase in pressure in both the thoracic and abdominal cavities, with the mouth open. The peak intra-abdominal pressure during a vomit is ~100–200 mmHg in both animals (e.g., cat [14], ferret [19]) and humans [20]. During both retching and vomiting, the abdominal muscles and diaphragm co-contract rhythmically [21,22].

The motor events of retching and vomiting (driven by phrenic and spinal motor neurones) and the preceding changes in gastrointestinal motility (vagally mediated proximal gastric relaxation and small intestine retrograde giant contraction) are coordinated from the brainstem (see [8,9] for review). The main brainstem nuclei involved in coordinating the emetic motor response are the nucleus tractus solitarius (NTS) in the dorsal brainstem and the connected nuclei in the ventral brainstem, comprising the ‘central pattern generator’ and the ventral respiratory group [16,23,24,25]. The NTS is activated to induce emesis by three main inputs:

(a) The area postrema. An area in the caudal extremity of the fourth ventricle where the blood–brain barrier is relatively permeable, providing a site at which circulating substances can act (e.g., dopamine D_2_ receptor agonists, µ opioid receptor agonists, GLP-1 receptor agonists and the cytokine GDF15; see [8,15,26,27] for references);

(b) The vestibular system. The output relays via the vestibular nuclei for the induction of emesis in response to aberrant body motion [15];

(c) Abdominal vagal afferents. Afferents terminating in the muscular wall of the digestive tract signal tension (‘mechanoreceptors’). The afferents terminating in the epithelium are responsive to endogenous neuroactive agents (e.g., 5-hydroxytrytamine, substance P, cholecystokinin) released from enteroendocrine cells (EEC) by luminal chemical (e.g., specific nutrients, vomitoxin, Staphylococcal enterotoxins, copper sulphate, hypertonic solutions) or mechanical stimuli. Systemic stimuli (e.g., the cytotoxic anti-cancer drug cisplatin) can also induce the release of neuroactive agents from the EECs [8,9,15,28]. The emetic response to stimuli acting through this pathway is acutely blocked and/or diminished by abdominal vagotomy [28].

For recent reviews of emetic mechanisms see [8,9,15,27,29,30].

Contrary to the current prevailing view that rodents lack the ability to retch or vomit, there are some publications which report ‘retching’ in mice [31,32,33,34]. Retching can be considered as ‘non-productive’ vomiting (i.e., without forceful oral expulsion of gastric contents). Recently, there have been three additional published studies on mice, reporting the presence of either ‘retching-like behaviour’ [35,36] or ‘vomiting of liquid’ [37].

In view of the extensive use of rodents in research, including toxicology, e.g., [38,39,40,41], and the potential broader biological implications if rodents are able to vomit (see Section 6), we undertook a critical assessment of the evidence supporting or refuting the ability of rats, mice and other rodents to vomit and/or retch. The use of rodents to study ‘nausea’ is not discussed here. The reader is referred to the following examples of rodent ‘nausea’ studies [42,43,44,45] and reviews discussing some of the limitations: especially those relating to the uncertain translation of ‘nausea-like’ behaviours in rodents to the experience of nausea in humans [9,15].

## 2. Search Methodology and Aims of Review

To identify the relevant publications, the following approaches were used:

(a) Databases (e.g., PubMed) were searched using the following terms: rodent AND retching/vomiting/emesis; mouse AND retching/vomiting/emesis; rat AND retching/vomiting/emesis; guinea pig AND retching/vomiting/emesis; and rabbit AND retching/vomiting/emesis. We also searched gag reflex AND rodent/rat/mouse. Publications were carefully examined, as although the title may imply that retching or vomiting was observed, this may not be the case. For example, a paper entitled “*Arcuate nucleus orexin-A signalling alleviates cisplatin-induced nausea and vomiting through the paraventricular nucleus of the hypothalamus in rats*” [46] reports data on kaolin consumption and gastric motility but not vomiting. Similarly, a paper entitled “*Relationship between analgesic dose of morphine and vomiting in rat model of postoperative acute pain*” reports data on grimacing (considered to be a sign of pain) and kaolin intake but not vomiting [47].

(b) Reference lists of publications on this topic with which the authors were already familiar were searched to identify earlier relevant publications.

(c) As the most recent publications originated from China and some earlier ones from Japan, related publications that were not in the English language were translated.

The specific aims of this narrative review are as follows:

(a) Review the literature on the absence and presence of emesis in rodents and critically analyse the findings;

(b) Attempt to reconcile the ‘historic’ and ‘recent’ observations through discussion of morphological, physiological and evolutionary differences between rodents and species with a defined emetic reflex that may explain their unusual emetic reflex;

(c) Consider the implications of these findings for the use of rodents in aspects of biological and biomedical research [4,39,48] and for understanding the evolution of the vomiting reflex in mammals.

## 3. A Historical Perspective on the Reported Inability of Rodents to Vomit

Firstly, we review publications in which emesis was not reported in rats, mice or other rodents (e.g., guinea pig) following the administration of a drug or exposure to a stimulus (motion) known to be emetic in other species. Secondly, to provide more direct ‘evidence of absence rather than absence of evidence’, we review studies where the potential ability of rodents to retch or vomit was investigated directly.

### 3.1. Circumstantial Evidence for the Absence of Emesis in Rodents

The earliest publication discussing this issue that we are aware of is from 1923 [2], in a detailed paper entitled ‘Studies on vomiting’. They noted, “*text-books commonly attribute the inability of solipeds, ruminants, rodents and others to vomit to the position of the stomach; to the marked development of the fundus; or to the length of esophagus below the diaphragm*”. Throughout the manuscript, we use direct quotations from the original publication to accurately reflect either the prevalent view at the time or the event reported by the authors upon which we comment. That the inability of rodents to vomit continued to be the accepted view is supported by a comment in a 1955 paper noting retching and vomiting in response to protoveratrine in the guinea pig: “*So far as we are aware, this is the* *first time* (our underline) *that vomiting has been described in rodents*” [49]. A review of the phylogenetic aspects of vomiting in 1971 [50] regarding rats states “*never vomits; stops eating*” and similar comments are found in the current literature regarding mice; for example, “*Assessment of MS* (motion sickness, our insert) *in mice is hindered by the lack of emetic reflex and difficulty to unequivocally identify nausea*” [51], p. 2.

The above comments illustrate the prevalent view of the inability of rodents to vomit, reported over ~100 years. Prior to the use of the insectivore *Suncus murinus* (house musk shrew) in laboratory studies of emesis [3,4], ferrets [19], cats and dogs, all carnivores, were the most commonly used species [7,8,11,52]. This was despite the availability of rats and mice, consistent with the assumption that the latter could not be used for such studies. Indeed, rats have been exposed to a very wide range of stimuli which induce emesis in other species, in part because they exhibit pica (assessed in laboratory studies by the consumption of kaolin) and conditioned tase aversion (see [4,9,15,53]). However, we have not identified reports of retching or vomiting in these types of study, thus providing circumstantial evidence for the absence of emesis.

More direct evidence for the absence of emesis in rodents comes from studies of regional skin temperature (e.g., increase in tail temperature) monitored with thermal imaging cameras in rats and mice before, during and after rotation. In these studies, retching and vomiting was not reported and the experimental methodology for observing the animals makes it unlikely that it would have been missed [51,54,55,56,57].

Rats and mice are, and have been, used extensively by the pharmaceutical industry for both basic research and toxicology [41]. Although much data is not published, it seems highly unlikely that if retching or vomiting were reliably observed in rodents that it would not have been reported within a published safety study or regulatory report. This is especially so when central nervous system toxicity is investigated [38,40,41,58]. Additionally, there are examples of pharmaceutical company publications where both a rodent and an emetic species have been studied by using the same compounds without reports of emesis in the rodent (e.g., [59], rat and ferret using apomorphine and ABT-594, an α4β2neuronal nicotinic receptor agonist; [60], rat and shrew, DSP-1053, a serotonin uptake inhibitor with 5-HT_1A_ receptor partial agonist activity; [61], rat, ferret and dog, apomorphine and rat and ferret, cisplatin).

Finally, comparative physiology [62] and veterinary medicine textbooks classically regard rodents as being unable to vomit (e.g., [63,64,65]). This has clinical implications, such as contraindication for the use of emetics [64] or precluding the need for pre-operative fasting (e.g., [65]).

### 3.2. Direct Evidence for the Absence of Emesis in Rodents

An investigation of the assumption that rodents are unable to vomit studied the behavioural responses of six species of rodent (including rat) to emetic stimuli (subcutaneous [s.c.] apomorphine, s.c. veratrine and intragastric [i.g.] copper sulphate) [3]. Neither retching nor vomiting were observed in any of the rodent species in response to any of the stimuli.

Using the *in situ* perfused brainstem preparation [66] with resiniferatoxin or abdominal vagal afferent electrical stimulation as emetic stimuli, there was no evidence for an ‘emetic-like’ response in either rat (Sprague Dawley) or mouse (C57BL/6), as indicated by oesophageal shortening, efferent phrenic nerve activity and measurement of mouth movements [3]. In the same preparation, an ‘emetic-like’ response was evoked in the house musk shrew [3]. Thus, the above study [3] provides the most direct evidence for the lack of retching and vomiting in rats and mice, as well as in a number of other rodents including guinea pig (*Cavia porcella*), mountain beaver (*Aplodontia rufa*; a type of ground squirrel), Townsend’s vole (*Microtus townsendii*) and nutria (*Myocaster coypus*).

In other experiments with rats, using intragastric hypertonic sodium chloride, known to be emetic [67], and veratridine, gastric emptying was delayed [53] and the number of chewing movements per hour increased [4]. However, no retching or vomiting was observed within an hour of dosing, as would have occurred in an emetic species.

These findings in the rat are consistent with a comment in 1989 by Borison [26] (p. 365), who was widely recognised as a leading expert in the field at the time [68]: “*in our laboratory, we were unable with the use of intra-thoracic pressure recording to detect any effort made by rats to retch or vomit in response to a variety of emetic drugs and so we could find no objective evidence to support the suggested mechanism of mechanically frustrated vomiting (unpublished observations).*”

To summarise, there is a wealth of circumstantial evidence that rodents, primarily exemplified by rats, are unable to vomit, and with the exception of the studies discussed below in Section 4, we have found no other evidence for retching.

## 4. Analysis of Reports of Retching and Vomiting in Rodents from 1920 to 2025

**Figure 1:**
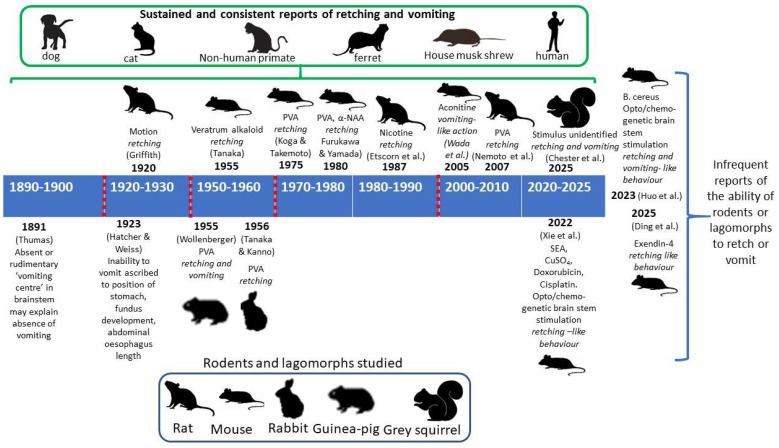
A summary of selected publications with dates (in bold), commenting or reporting data on retching and/or vomiting (response is given in italics) in rodents, together with the stimuli used. The terms ‘retching’ and ‘vomiting’ are as described in the original publication but see text for discussion of terminology and data interpretation. Further details of the studies are given in the text and/or Table 1, Table 2 and Table 3. References: Chester et al., 2025 [69]; Ding et al., 2025 [36]; Etscorn et al., [70]; Furakawa & Yamada, 1980 [31]; Griffith, 1920 [71]; Hatcher & Weiss, 1923 [2]; Huo et al., 2023 [37]; Koga & Takemoto, 1975 [32]; Nemoto et al., 2007 [72]; Tanaka, 1955 [33]; Tanaka & Kanno, 1956 [73]; Thumas, 1891 [74]; Wada et al., 2005 [75]; Xie et al., 2022 [35]. α-NAA = α-naphthoxyacetic acid; CuSO_4_ = copper sulphate; PVA = protoveratrine-A; and SEA = *Staphylococcus aureus* A toxin. Note that the number of publications reporting ‘retching or vomiting’ over >100 years are scattered and very small in number in contrast to the sustained and consistent publications on retching and vomiting in dogs, cats, non-human primates and humans and, more recently ferrets, house musk and shrews [2,3,4,7,11,27].

This section reviews the historical studies on mice (Table 1) and other rodents (Table 2) before analysing the recent reports of retching and/or vomiting [35,36,37]. As lagomorphs are also reported to be unable to vomit [2,3], we comment on publications noting ‘retching’ in rabbits (*Oryctolagus cuniculus)*. Figure 1 summarises the dates of the key publications reporting ‘emesis’ in rodents and lagomorphs over the last 100+ years.

**Table 1:** A summary of historic data (chronological order) on “retching-like” behaviour in mice, as defined in the original publications. See text for further details.

Stimulus/Route	Response	Latency	Number of R-like Events; N = Respond/Tested	Ref
Veratrum ester alkaloids, s.c.	“*movement of head and front part of the body as in the vomiting act*”; described as “retching movement”	NS	R = NS N = 5–10/dose (usually 8)	[33]
Protoveratrine-A_,_ s.c.	No description other than “*retching*”	10–15 min	Respond/tested = Almost 100% in high dose group	[32]
Protoveratrine-A, i.p.	“*downward and quick opening of the mouth, with upward and downward movement of the thoracic skin”; described as* “retching” accompanied by salivation, preening and decrease in exploratory behaviour	NS	R = 11.6 ± 3.8 in 60 min. N = 10/10	[31]
α-naphthoxyacetic acid, i.p.	“*recurrent episodes of wide opening of the mouth*”; described as “retching” accompanied by salivation, lacrimation and sometimes a sudden jump	5 min	R = 15.8 ± 4.2 in 30 min N = 10/10	[31]
Aconitine, p.o.	“Vomiting-like action” with mouth opening	3–15 min	NS	[75]
Protoveratrine-A, s.c.	‘Retching-like behaviour’, mouth opening but also a separate ‘hiccup-like’ behaviour	10–15 min	R = 14.3 (mean) -; 34.9 in 60 min (dose-related; N = 4)	[72]

Abbreviations: i.p. = intraperitoneal; NS = data not stated in the publication; R = number of “retching-like” events as defined by the authors; and s.c. = subcutaneous.

**Table 2:** A summary of data of ‘retching-like’ behaviour in rats, guinea pigs and rabbits, as defined in the original publication. See text for further details.

Species	‘Emetic’ Stimulus (Dose/Route)	Description of Response	Latency	Ref.
RAT	Horizontal rotation	“*Frequent and severe retching movements accompanied by defecation and micturition*”	NS	[71]
RAT	“*Poisoned*”	“*The abdomen retches, the back arches, the head lowers, the mouth gapes, and the tongue protrudes*”; described as “*Behavioral indices of emesis*”	NS	[76]
RAT	Nicotine (0.5–3.0 mg/kg; i.p.)	“*occasional retching*” in all dose groups	NS	[70]
RAT	Protoveratrine-A (50–200 µg/kg; s.c.)	‘Retching’, tongue protrusion, mouth opening downward, forelimb straddling	10–15 min	[72]
GUINEA PIG	Protoveratrine (60% A and 40% B; 150 µg/kg; i.p.)	“*violent retching movements were noticed, which were followed by vomiting*”………… “*The hind legs became immobilized in flaccid paralysis while vomiting continued*”	A few minutes	[49]
GUINEA PIG	Copper sulphate (i.g.)	“*discomfort symptoms similar to vomiting*”. Gastrointestinal tract electrical activity was recorded, with the authors reporting “disturbances” (increased frequency and amplitude) coinciding with the “symptoms similar to vomiting” *	3.7 min	[77]
RABBIT	Protoveratrine-A (10 µg/kg; i.v.)	*Retching act: “movement of the head and front part of the body as in the vomiting act……. Masticating movements of mouth, licking the lips, marked salivation, dyspnoea and cyanosis were always associated with the retching period*”	1–2 min	[73]
RABBIT	Apomorphine	No description; anecdotal report of ‘retching’	NS	[78]
GREY SQUIRREL	‘Spontaneous’—stimulus not identified	Retching defined as “*attempting to vomit without bringing anything up*” [79]	NS	[69]

Abbreviations: i.g. = intragastric; i.p. = intraperitoneal; i.v. = intravenous; NS = data not stated in the publication; s.c. = subcutaneous; and * = description from a translation of the original paper.

### 4.1. Historical Reports

(a) Rats and mice. Three publications using mice report ‘retching’ induced by veratrum alkaloids (s.c. or i.p.), including protoveratrine-A and an additional report using α-NOAA (Table 1). The widely used emetic agent apomorphine (0.05–2 mg/kg. i.p., a dopamine D_2_/D_3_ receptor agonist), acting via the area postrema [26], did not induce ‘retching’ in mice [31]. However, this negative finding should be treated with caution, as even amongst species with a defined emetic reflex, there is a wide variation in the sensitivity to apomorphine [7,80,81] and ‘bell shaped’ dose–response curves are observed for the emetic response in ferrets [52]. Publication [31] reports that ‘retching’ was accompanied by opening of the mouth and salivation, but the earliest report we found in mice [33] only describes movements of the head and front part of the body “*as in the vomiting act*”. In addition, we identified rat publications describing “*frequent and severe retching movements*” induced by horizontal rotation [71] or “*abdominal retching*” accompanied by back arching, lowering of the head, mouth gaping and tongue protrusion in response to an unspecified poison [76]. The early studies of veratrum alkaloid-induced retching in the mouse (Table 1) are consistent with a later mouse study of the related agent aconitine [75]. The mouse veratrum alkaloid studies were replicated and extended to the rat [72] (Table 2). The response in the mouse to PVA was inhibited by a neurokinin_1_ receptor antagonist (NK_1_ RA, CP-99,994, 20 mg/kg, s.c.) or the 5-hydroxytryptamine_3/4_ receptor antagonist indisetron (5 mg/kg, s.c.) [72].

Describing the rejection response to flavours paired with illness in a rat, it is reported that “*It gapes, retches, and rubs its chin on the floor*” Garcia, 1981, cited in [82]. However, others who have studied the rejection behaviour in the rat do not report retching, but do report mouth gaping [83,84].

The mechanical events of a retch and a gag are similar [10]. The gag reflex is present in rats [85,86] and mice [87], defending the pharynx and respiratory systems through pharyngeal/velar constriction [88]. Externally, in the rat, a gag is described as “*simultaneous wide jaw excursion, vigorous anterior tongue protrusion, abdominal contraction and thoracic fixation*” [86]. As noted in [88], severe gagging is a combination of “*gagging and some aspect of the emetic response*.” The gag reflex is normally induced by mechanical probing of the oropharynx, activating the superior and recurrent laryngeal nerve afferents. Experimentally, high-frequency (20–50 Hz, 1 s) electrical stimulation of the superior laryngeal nerve afferents in the decerebrate rat evoked ‘severe gagging’ (the authors description), which was characterised by a single burst of increased abdominal muscle EMG activity, lasting ~250 ms, with a coincident burst of activity of the costal diaphragm but inactivity of the crural portion [89]. Pressure changes in the oesophagus accompanied the gagging, with the pressure rising first in the lower oesophagus and then in the upper, suggesting progression of a retrograde contraction. These physiological features of ‘gagging’ are relevant to understanding the scattered reports of retching-like behaviour in rodents discussed below.

(b) Other rodents and lagomorphs (Table 2). In the guinea pig, retching followed by ‘vomiting’ in response to protoveratrine is described [49]. Also, in the guinea pig, Ref. [77] “*discomfort symptoms similar to vomiting*” are reported in response to intra-gastric copper sulphate but the description is insufficient to draw a clear conclusion. The woodchuck (*Marmota monax*) “*may vomit from red squill*” (Table 1, [45]), but no reference was given (red squill has been used as a rodenticide—see below [90]). In the rabbit [73], protoveratrine-A (10 µg/kg, i.v.) induced “*movement of head and front part of the body as in the vomiting act*”, and in the more severe instances, the animals “*put the forelimbs on the mouth standing on hindlimbs, and acted the retching movement with ejaculatory phonation*”. This apparent ‘retching’ was accompanied by salivation, lip licking, masticating movements, dyspnoea and cyanosis. Most recently, spontaneous episodes of retching (2–9 retches each lasting ~1 s) followed by expulsion of white/brown liquid were fortuitously recorded in a wild grey squirrel (*Sciurus carolinensis*) [69]. These episodes were accompanied by a chewing motion of the jaw.

### 4.2. Recent Mouse Studies Reporting ‘Retching-like Behaviour’ (Table 3)

The above studies relied solely on observation of the external appearance of the animal to conclude that retching or vomiting occurred. However, two recent studies [35,37] additionally used physiological measures (diaphragm or abdominal muscle electromyogram, intragastric pressure), making it possible to compare with similar studies on emetic species (see Introduction). A third paper [36] made behavioural observations. In this section, we only concern ourselves with the data that are directly relevant to ‘retching-like’ behaviour but the publications also report detailed data on the molecular biology of the brainstem pathways implicated.

Xie et al., 2022 [35] showed that wide mouth opening (large amplitude and long duration recorded with a high-speed camera), distinguishable from gaping induced by bitter tasting substances, was induced by Staphylococcal enterotoxin A (SEA; 0.1 and 0.3 mg/kg, i.p.) in conscious animals. The wide mouth opening was defined by the authors as a ‘retching-like’ behaviour, with the number of events being quantified and the total mouth opening time being reported as ‘time for retching’ during a specified observation period. The physiological correlate of this mouth-opening behaviour was studied in animals given SEA and in which the EMG activity was recorded from the diaphragm (right dome) and abdominal muscles (external oblique). Figure 1D in [35] shows five discrete bursts of synchronous EMG activity of both muscles when the mouth was open for ~2 s, in contrast to their EMG activity when the mouth was closed, which was less intense and desynchronised (diaphragm before abdominal activity). With a dose of 0.3 mg/kg SEA (Table S1 in [35]), the magnitude of the diaphragm EMG change was 314 ± 34% and the abdominal EMG change was 287 ± 25%. Although Figure 1D in [35] shows an example when the mouth was open for ~2 s, the time the mouth was wide open was usually shorter, with a mean time of 1.063 ± 0.063 s, ~5× longer than spontaneous mouth opening (0.185 ± 0.007 s) ([35] Table S1 and Figure S1).

It should be noted that the total time spent demonstrating this ‘retching-like’ behaviour during a 180 min observation was dose-related but was only 4–8 s at the highest dose of SEA studied (0.3 mg/kg, i.p; Figure 1G, [35]). ‘Retching-like’ behaviour was also induced by Staphylococcal enterotoxins B, C1, D, E and H) (Table 3). The ‘retching-like’ behaviour (‘time for retching’) to SEA was markedly reduced by chronic abdominal vagotomy and by an NK_1_ RA (CP-99,994, 10 mg/kg, i.p.) and a 5-hydroxytryptamine_3_ receptor antagonist (5-HT_3_ RA, granisetron, 5 mg.kg, i.p.), which was consistent with studies showing that this toxin acts via the enteroendocrine cell–abdominal vagal afferent pathway in emetic species [91].

Using video recording of mouth-opening time, this study also showed that protoveratrine-A and intragastric copper sulphate (widely used in older studies of emesis, e.g., [7,11]) induced ‘retching-like behaviour’ (Table 3). Additionally, the cytotoxic anti-cancer drug cisplatin (a highly efficacious emetic in humans and animal models such as the ferret [19]) “*only slightly evoked*” retching-like behaviour, whereas doxorubicin (another cytotoxic drug) was “*more effective*” at inducing retching-like behaviour. With all the above stimuli, the time spent demonstrating ‘retching-like’ behaviour was relatively brief, ranging from ~1 to ~10 s (Table 3).

**Table 3:** Summary of data in Xie et al., 2022 [35], Huo et al., 2024 [37] and Ding et al., 2025 [36].

Stimulus/Dose/Route	Response to Stimulus	Latency	Number of R-like or V-like Events	Duration	Ref.
* SEA; 0.3 mg/kg; i.p.	“Retching-like behavior”: wide mouth opening (video camera) and when measured associated with synchronous bursts of EMG activity from diaphragm and abdominal external oblique muscles	87 ± 14 min	R = 5 ± 3 in 180 min; N = 9	~6 s	[35]
** PV-A; 0.4 mg/kg; i.p.	“Retching-like behavior” (VR)	NS	R = NS N = 9	~6 s	[35]
** CuSO_4_; 120 mg/kg; i.g.	“Retching-like behavior” (VR)	NS	R = NS N = 9	~1 s	[35]
** Doxorubicin; 10 mg/kg; i.p.	“Retching-like behavior” (VR)	NS	R = NS N = 7	~6 s	[35]
** Cisplatin; 10 mg/kg; i.p.	“Retching-like behavior” (VR)	NS	R = NS N = 7	~5 s	[35]
** Optogenetic activation of Tac1^+^ DVC neurons; 10 mW, 20 Hz	“Retching-like behavior” (VR)	NS but ‘Immediate’ from records	R = NS N = 9	~6 s	[35]
** Chemogenetic activation of Tac1^+^ DVC neurons by SEA; i.p.	“Retching-like behavior” (VR)	88 ± 13 min	R = NS N = 9	~10 s	[35]
** Chemogenetic activation of Tac1^+^ DVC neurons	“Retching-like behavior” (VR)	15 ± 2 min	R = NS N = 9	~20 s	[35]
** *B. cereus*; 10 µL/g; i.g.; hourly for 3 h.	“Retching-like behavior”: wide mouth-opening angle (VR) and where measured, a simultaneous transient increase in IGP ± diaphragm EMG	~60 min	R = 5–29 in 3 h N = 10	NS	[37]
** Optogenetic activation of glutamatergic NTS neurons; 20 mW; 5 s	“Retching-like behavior”: wide mouth-opening angle (VR)	NS but “Immediate” from records	R = ~4 in 5 s N = 4	NS	[37]
** Optogenetic activation of Calb1^+^ NTS neurons; 20 mW, 20 Hz, 5 s.	“Retching-like behavior”: wide mouth-opening angle (VR). Also, 10 Hz stimulation induced 2 mouth-opening events and a rise in IGP (<0.2 mmHg) in 5 s.	NS but “Immediate” from records	R = ~4 in 5 s N = 4	NS	[37]
** Optogenetic stimulation of NTS-Amb-RVLM pathway; 20 mW, 20 Hz	“Retching-like behavior” (VR). In a group of 4 animals, stimulation of the same pathway at 10 Hz for 1 sec evoked a single rise in IGP (<0.2 mmHg), coincidental with wide mouth gaping and increased diaphragm EMG activity.	NS but “Immediate” from records	R = ~4 in 5 s N = 18	NS	[37]
** Chemogenetic stimulation of *B. cereus* TRAPed NTS neurons; i.g.	“Retching-like behavior”: wide mouth-opening angle (VR) and where measured, a simultaneous transient increase in IGP	NS	R = ~40 in 60 min. N = 5	NS	[37]
Optogenetic stimulation of NTS-Amb-RVLM pathway; 20 mW, 20 Hz	Repeated activation evoked “*forceful vomiting*”—oral ejection of gavaged dragon fruit juice	NS	V = Occurred and vomitus weighed N = 6	NS	[37]
Exendin-4; 1–1000 µg/kg; i.p.	“Retching-like behavior”: “unusual wide mouth-opening movements”	~80 min (1, 10, 100 µg/kg); ~50 min (1000 µg/kg)	1 µg/kg: R = ~4 in 3 h, (~3–5), N = 10 10 µg/kg: R = ~6 in 3 h (~5–7), N = 10 100 µg/kg: R = ~6 in 3 h (~4–8), N = 10 1000 µg/kg: R = ~7 in 3 h (~6–8), N = 10	NS	[36]

* Only data with the highest dose of SEA is shown in this table and the authors also studied other Staphylococcal enterotoxins (see text). ** Also studied at other doses, stimulus parameters (optogenetic laser stimulation only) or route. The response shown here is the one of greatest magnitude with that stimulus. ^+^ The data on ‘duration’ is derived from the histograms in the figures and is the “*total time spent in retching-like behavior in each mouse calculated by adding the time spent in each mouth-opening action*” ([35] e4). Data on the duration of each mouth-opening action is given in [35], Figure S1 and in Table S1, but is labelled in the table as “Retching duration”. In Ding et al. [36], data are estimated from figures. For further details, see text. Abbreviations: Amb = nucleus ambiguus; CuSO_4_ = copper sulphate; EMG = electromyography recording from either the diaphragm or abdominal muscles; i.g. = intragastric/gavage; IGP = intra-gastric pressure; i.p. = intraperitoneal; N = number of animals; NS = not stated; NTS = nucleus tractus solitarius; PV-A = protoveratrine A; R = retches; RVLM = rostro-ventrolateral medulla; SEA = Staphylococcal enterotoxin A; V = vomits; and VR = data collected by video recording.

‘Retching-like behaviour’ was also evoked using optogenetic and chemogenetic activation of tachykininergic neurones in the brainstem dorsal vagal complex (NTS, area postrema and dorsal motor vagal nucleus), with synchronised bursts of EMG activity of the diaphragm and abdominal muscles accompanying the mouth opening [35].

Huo et al., 2024 [37] extends the range of substances inducing ‘retching-like’ behaviour in mice by using *Bacillus cereus* (i.g.) and its toxin cerulide in animals with experimentally induced gastritis (i.g. aspirin and hydrochloric acid; [37], Supplementary Methods). Although in most studies, the ‘retching-like’ behaviour was measured by the number of wide mouth openings, some studies also monitored intra-gastric pressure (IGP, via an implanted latex balloon and cannula) and diaphragm (left costal region) EMG activity. An additional novel aspect was the use of orally gavaged dragon fruit juice to identify ‘vomiting’ in response to optogenetic brainstem stimulation. Below, we review the evidence provided by each recording technique for the presence of ‘retching-like’ behaviour and ‘vomiting,’ as defined by the authors.

(i) Mouth gaping, intra-gastric pressure and diaphragm EMG. In response to *B. cereus* (i.g., hourly for 3 h), episodes of jaw gaping and a transient increase in IGP occurred. These events were temporally correlated (average correlation 0.6) and followed a similar time course of ~0.5 s, with each event defined as being ‘retching-like’ behaviour. A latency of the ‘retching-like’ response to *B. cereus* of ~1 h is reported (Supplementary Methods [37] p. 8) and over a 3 h period, the average number was ~15. The peak magnitude of the transient increase in intra-gastric pressure was very small (<0.2 mmHg).

Differences in the magnitude of the response to various stimuli are apparent (Table 3). For example, in *B. cereus*-treated *FosCreER* mice, ~10 episodes were reported in 3 h, but when chemogenetic stimulation (CNO 1 mg/kg) was used to activate the NTS neurones expressing hM3Dq-mCherry, an average of almost 40 ‘retches’ was observed in 1 h.

Optogenetic stimulation of the Calb1NTS-Amb/RVLM pathway for 1 s evoked a single ‘retch’, which was characterised by a transient increase in IGP (~0.2 mmHg), correlated with an increase in diaphragm EMG activity and gaping. Analysis of the EMG showed an increase of three times over the baseline.

(ii) Dragon fruit juice study. Optogenetic stimulation of the NTS, Amb/RVLM pathway for 20 s in Calb1^+^ mice evoked oral expulsion of intra-gastric dragon fruit juice (given by gavage 10 min before stimulation), which the authors defined as “*forceful vomiting*”. The video ([37] S6) shows the ejection of fluid but it appears to be more reminiscent of passive dropping from the mouth following regurgitation, rather than the active ejection that would occur during vomiting, especially with liquids. Although the expelled fluid is coloured, this could arise from residual dragon fruit juice in the oesophagus following gavage; a contribution of increased salivation cannot be excluded. The brain area stimulated included the nucleus ambiguus, which provides the motor innervation for the pharynx and oesophagus [92,93], and thus could potentially cause gagging and regurgitation, which could be confused with ‘vomiting’.

In rats and mice, it is advised that the gavaged volume should not exceed 1% of body weight or 10 mL/kg in rats and mice [94]. In [37], p. 14, if we assume a body weight of 25–35 g for the mice, then 2.5 mL/mouse is 7–10% of the body weight or 70–100 mL/kg. Reflux of gastric contents is associated with gavage in the rat [92] and is more likely to occur at higher volumes.

Ding et al., 2025 [36] is the most recent study, and it reported the induction of “*retching-like behavior*” by the GLP-1 receptor agonist exendin-4 (i.p., Table 3), using what the authors described as “…*a recently established murine model of emesis, where wide-mouth opening serves as a behavioral proxy for retching-like behavior*” [37], p. E254. Injection of exendin-4 directly into the area postrema rapidly induced the ‘retching-like behaviour,’ leading to the conclusion that this was the site of action, as is the case in emetic species.

### 4.3. Summary of Historic and Recent Rodent Studies

(i) The consensus view from the literature is that rodents and lagomorphs do not have an emetic reflex (especially vomiting), which is explained by behavioural, physiological and anatomical differences from emetic species (e.g., [3,4,9]). The evidence is from the following: (a) direct investigation of the ability of selected rodents to retch/vomit (e.g., [3]); and (b) studies using close observation for an appropriate time in which a drug was administered at least at doses known to be emetic in other species, or the animal was exposed to abnormal motion and neither retching or vomiting was reported (Section 3.1 and Section 3.2 for details and references).

(ii) Using direct observation, ‘retching’, ‘retching-like behaviour’ or ‘retching movements’ have been reported as an incidental finding in a few studies of mice, rats, guinea pigs and rabbits over the last 100 years (Table 1 and Table 2; Supplementary Tables S1 and S2 with additional experimental details).

(iii) The recent mouse studies (Table 3; Supplementary Table S3 with additional experimental details) showed that the ‘wide mouth-open behaviour’ is accompanied by synchronised bursts of increased EMG activity (~3× baseline) of the costal diaphragm and abdominal muscle, and a very small increase (~0.2 mmHg) in intragastric pressure (Section 4.2). They also showed that this ‘retching-like’ behaviour can be evoked by various emetic substances, but the magnitude of the responses is generally small, being only more robust when direct optogenetic and chemogenetic stimulation of central pathways are used.

(iv) Objective evidence for ‘vomiting’ is much less than for retching (Section 4.1 and Section 4.2; Table 2). Huo et al. [37] demonstrated oral expulsion (not forceful) of dragon fruit juice following repeated optogenetic stimulation of the brainstem nuclei, and oral expulsion of white and brown contents after ‘retches’ is reported in the grey squirrel [69].

(v) Studies reporting any type of emetic behaviour in mice or rats are relatively few but are not confined to one strain, sex or country. Protoveratine-A is the pharmacological stimulus investigated in the widest range of rodent species (Table 1 and Table 2).

## 5. Critical Reappraisal of Retching and Vomiting in Rodents

### 5.1. What Is Being Observed in the Recent and Historic Papers Reporting Emesis in Rodents?

(a) Description of the events. The descriptions of thoracic contraction and posture change are consistent with the retching described in emetic species. However, describing a wide-open mouth and tongue protrusion as ‘retching-like’ behaviour is questionable, as in emetic species, these behaviours are typically observed during vomiting, while during retches, the mouth is usually closed or at least not gaping open [10,11,12,14,26]. These differences raise the question of how similar this ‘retching-like’ behaviour actually is to true retching in emetic species.

Retching is usually quantified by counting the individual number of retches (e.g., [10]). In the mouse, quantification of the effect of emetics was by “*time spent retching*”, i.e., the time spent with the mouth wide open. For the chemical emetics (Table 3 and Table S3), the total time spent demonstrating ‘*retching-like behavior*’ in the Xie et al. [35] study was ~1 to ~6 s, meaning that either each ‘retch’ was very short in duration or just a few episodes of this mouth-opening occurred. In *Suncus murinus*, an insectivore with juveniles of a similar weight to adult mice, a frequency of four retches/s and four to seven retches per burst are reported [95], while in the ferret, six to nine retches per burst and a frequency of one/s are reported [10], with similar values being reported for dogs and cats [10,95]. If the behaviours observed in mice were truly retches, the time spent retching would be expected to be much longer.

Figure 1B in reference [35] shows a range of three to five ‘retches’ over 3 h in the ten mice given SEA (0.3 mg/kg i.p.). The study reported in [37] counted the individual number of mouth-opening actions in response to *B. cereus*, with an average number of ~15 ‘retches’ in 3 h, which is again low when compared to responses to emetics in classical emetic species. Quantitatively, therefore, the mouth-opening behaviour does not appear to be comparable to retching in an emetic species.

However, the intense optogenetic stimulation of the NTS or the Amb/RVLM pathway evoked five ‘retches’ within 5 s ([37] Table 3), which is more compatible with what would be expected for true retching. This suggests a possible stimulus intensity effect to explain the relatively low magnitude of the response to natural emetic stimuli in comparison to the higher magnitude in response to direct central stimulation of the emetic pathways. Additionally, in the grey squirrel [69], several retching episodes (e.g., five times over 6 s) were reported, suggesting that several rodent species are able to ‘retch’ (see Section 5.3 and Section 6 for further discussions).

(b) Characteristics. The EMG and intragastric pressure recordings during ‘retching-like’ behaviour in the recent papers [35,37] provide additional data to question the conclusion that the behaviour reported is analogous to retching in emetic species.

Firstly, the synchronous increase in the costal diaphragm and abdominal muscles’ EMG activity is consistent with retching (and vomiting), but the magnitude of the change in the diaphragm EMG bursts when the mouth is open (‘retching’) as opposed to when it is closed (normal respiration) is ~3× in both studies (e.g., [35], Figure 1E; [37], Figure S8). This is a smaller relative change than would be expected from comparable recordings in emetic species (see also Section 5.3 below). The diaphragm is generally less muscular in rodents and lagomorphs, partially due to the presence of a larger central tendon [3]. Absolute animal size is not an issue with regards to emesis, as some shrew species are smaller than mice (*Sorex unguiculatus*, 9–12 g, [96]; *Cryptotis parva*, 5–6 g [97]) yet have the ability to retch and vomit, and studies on juvenile *Suncus murinus* showed that those with a body weight of 13–16 g were already responsive to emetic stimuli [98].

Secondly, in response to SEA, Xie et al. ([35], Figure 1D) showed that during episodes of mouth opening lasting ~2 s, there were five distinct bursts of abdominal and diaphragm EMG activity at a frequency ~1.4× higher than the baseline respiration rate. Such bursts are consistent with retching, but in an emetic species it would manifest as five retches instead of just a single prolonged mouth opening. In the behavioural ethograms in [35] (Figure 1B) for mice given SEA, and in the Supplementary Movies showing the ‘retching-like behaviour’ ([35], Movies S1–S3; [37]), it can be seen that the episodes of mouth opening are discrete and isolated. In contrast, retches in emetic species often occur rapidly, rhythmically and sequentially in bursts, ending in a single, forceful expulsive action (vomit) [10,14,95,99].

**Figure 2:**
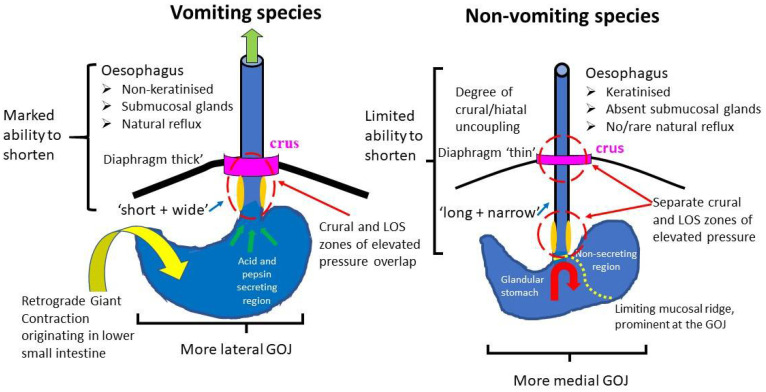
A diagram summarising the key morphological and physiological differences in the diaphragm, oesophagus, lower oesophageal sphincter (LOS), stomach and gastro-oesophageal junction (GOJ) between species with a well-defined ability to retch and vomit and rodents (see text for detailed discussion, caveats and references to supporting data).

Huo et al. [37] induced ‘retching-like behaviour’ with *B. cereus* and, as before, this was defined as wide opening of the mouth. Concomitant measurement of intragastric pressure showed a single, very small, pressure (~0.2 mmHg) rise with a similar time course to the mouth opening. The rise in intragastric pressure should reflect intra-abdominal pressure resulting from the contraction of the abdominal muscle and the diaphragm. However, the pressure is much smaller than might be expected when compared to that recorded during retching in species with a well-defined emetic response. For example, in anaesthetised adult *Suncus murinus* (body weight 65 ± 2 g), intra-abdominal pressure values of up to 15 mmHg were observed during retching ([95], Figure 1); in conscious animals, this value is likely to be higher because of the postural changes accompanying retching. In the decerebrate cat, intra-abdominal pressure waves of up to 60 mmHg are recorded ([14], Figure 2), comparable to ~70–100 mmg recorded in the conscious ferret [19]. The thoraco-abdominal muscles in mice can generate intrathoracic pressures of ~60 mmHg, as recently demonstrated in studies of ‘cough-like’ responses ([100], extended data Figure 3a), so the smaller magnitude of the intragastric pressure changes is probably not due to an incapacity to generate higher pressure gradients. The characteristics of the gastric balloon and the internal diameter of the cannula used to connect the balloon to the pressure transducer (via subcutaneous tunnelling to a connector on the head) could modify the fidelity of the pressure recording reported in [37]. Additionally, the IGP (~0.2 mmHg) recorded during ‘retching-like’ behaviour is smaller than the amplitude of the baseline gastric contractions recorded in mice *in vivo* [101] and *in vitro* [102].

The ‘retching-like behaviour’ in response to SEA in the mouse can be diminished by abdominal vagotomy ([35], Figure 1M), an NK1 RA (CP-99,994; Figure 1K) and a 5-HT3 RA (granisetron; Figure 1L). These findings are consistent with the current knowledge of toxins, such as SEA, inducing an emetic response by acting on the enteroendocrine cells to release 5-HT, which then activates abdominal vagal afferent 5-HT_3_ receptors and the involvement of brainstem NK_1_ receptors in mediating the emetic response [8,15,103,104]. In the decerebrate rat, ‘severe gagging’ induced by SLN stimulation was blocked by an NK_1_ RA (WIN51708) and an NMDA receptor antagonist (MK-801) given either i.v. [105] or microinjected into the brainstem [106]. These effects are consistent with studies of similar agents blocking retching/vomiting in emetic species [8,28,104] and providing indirect support that the ‘severe gagging’ in rats should be considered to be more like a retching response (i.e., truly an emetic-like response and not purely gagging). This is also consistent with the observation that the single gag induced by mechanical pharyngeal stimulation in an emetic species (ferret) was not blocked by an NK_1_ RA [107]. However, these comments should be treated with caution until further studies are undertaken (see below) to better characterise the ‘retching-like’/’gagging’ events in rodents, as qualitatively similar ‘cough like’ events in mice can be blocked by an NK_1_ RA [100]. Also, the potential of NK_1_ RAs to affect gastro-oesophageal reflux/regurgitation in rodents is not known, although the neuronal activity in the rat DMVN can be modulated by an NK_1_ RA [108].

### 5.2. Why Has Retching Not Been More Frequently Reported in Rodents?

If rodents do display ‘retching-like behaviour’, and may be able to expel liquid gastric contents (but not forcibly as it occurs in vomiting), then why has this not been reported more frequently? This is surprising, considering the number of rodents used in research globally and the number of potentially emetic toxins that have been administered to laboratory rodents in pharmaceutical company and academic research.

(a) Expectation. As it is widely assumed that rodents do not have an emetic reflex, there may be an expectation that the behaviours observed that may be classified as retching in other species are considered to be ‘another behaviour’ in rodents. For example, in mice with protoveratrine-A, ‘hiccup-like behaviour’ was observed, which was distinguishable from ‘retching-like behaviour’ (Table 1), and ‘laboured breathing’ was reported in guinea pigs in addition to ‘retching’ [49]. Retching-like behaviour could also be obscured by concomitant locomotor paralysis, spasms and laboured breathing (e.g., PVA, guinea pig, [49]) or wet dog shakes, ataxia and prostration (e.g., nicotine, rat, [70]). Further, the thoracic/abdominal movements may be confused with neurological effects of a test substance, particularly with agents like veratrum alkaloids that have widespread actions on voltage-gated Na^+^ channels. However, Horn et al. [3] carefully monitored behaviour in a range of rodents in response to several emetics over 40 min and neither retching nor vomiting occurred. Studies which do not report emesis in rodents have often described increases in licking, swallowing, chewing or salivation ([3,4]; see Section 3.2 above), which could be indicators of gastro-oesophageal reflux or regurgitation and hypersalivation could contribute to liquid appearing to drip from the mouth.

(b) The speed and magnitude of ‘retching-like behaviour’ in rodents. The video recordings in [37] and the quantitative analysis of ‘retching-like behaviour’ in [35,37] show that the events are brief (<1 s) and relatively infrequent, with the total time spent retching being <10 s for all chemical stimuli. The response could easily be missed by an observer, even if reviewing a video recording, unless played in slow motion. Without a close observation of the animals for a time that is sufficient for the ‘retching-like behaviour’ to manifest, it would be easy to obtain a false negative.

(c) Strain and species differences. It is conceivable that the strains of rats and mice in which an emetic-like response has been reported differ in some aspect of their physiology from those where a response has not been reported. We can find no evidence for this, but the small number of studies in which a response has been observed means that it is difficult to reach a firm conclusion. Notably, rodent supplier-dependent variations in the physiological responses to motilin have been invoked to explain the discrepancies in the literature [109], which is an observation of relevance to emesis because the motilin system is present in emetic species and absent in non-emetic species [110]. There is some evidence for strain differences in mice in the pica response to emetics [111,112], and even in the emetic species *Suncus murinus*, there are both strain and sex differences in emetic sensitivity [113,114]. Of particular relevance is that selective breeding of *Suncus murinus* has been shown to produce strains with marked differences in sensitivity to the emetic veratrine sulphate [115], related to PVA, which is used in the older reports of emetic responses in rodents (Table 1 and Table 2).

(d) Susceptibility to the stimulus. Even if we assume that what is observed in these studies is an emetic-like response, there may be issues with susceptibility to the stimulus. Thus, since it has been argued that rodents have ‘lost’ the ability to vomit (a behaviour present in progenitor mammalian species [110]), then it can be hypothesised that a degenerate or ‘pseudo’ emetic reflex is retained with a high threshold for activation. If so, then only stimuli causing direct intense activation and/or activating a specific input (e.g., vagal afferents, SEA, cisplatin) may be effective. Direct optogenetic and chemogenetic stimulation of the brainstem nuclei (NTS/Amb/RVLM) implicated in emesis seems to cause the most robust ‘retching-like behaviour’ (see Table 3, Table S3; [35,37]), together with veratrum alkaloids (Table 1 and Table 2, Tables S1 and S2), which can cause widespread activation of voltage-gated Na^+^ channels [116]. These observations suggest that stimuli having strong direct neuroactive effects are especially potent in revealing the hypothesised degenerate emetic reflex of rodents.

### 5.3. Are Rodents Capable of Vomiting, and If Not, Why Not?

Compared to retching, it should be possible to be more confident about observational reports of vomiting, but only if they describe what is expelled, the way in which this occurred (e.g., forcible and projected away from the body or dropping from the mouth) and ideally the pH, if it is liquid, to differentiate the gastric contents from saliva. Only two reports of ‘vomiting’ in rodents or lagomorphs describe the expelled material (mouse [37]; squirrel [69]). However, as mentioned previously, the oral expulsion of the gavaged fruit juice reported in [37] does not appear to be similar to true vomiting. So, what could be occurring? Gastric contents could access the mouth initially by reflux into the oesophagus, retroperistalsis in the oesophagus or simply by retrograde movement if sufficient fluid is gavaged into the stomach, with a final release from the mouth passively, and/or by the tongue or gagging. There is preliminary evidence requiring confirmation for oesophageal retrograde contraction associated with severe gagging in rats [89]. There would be little activity that is externally visible until just prior to ejection, when the mouth would open and the bolus would drop from the mouth. However, this is not vomiting, as the expulsion is not forceful and the mechanism does not involve diaphragm and abdominal muscle contraction. Therefore, the reported ‘vomiting’ of dragon fruit juice may be more akin to regurgitation, rather than true vomiting, but further study is required.

The recent report on a grey squirrel [69] describes motor activity which appears to be comparable to retching in species capable of vomiting, with each ‘retch’ lasting <1 s, and at an apparent frequency of 1/s, similar to the frequency reported, for example, in ferrets [10]. Following the ‘retches’, the squirrel expelled viscous white and brown liquid. However, it does so rather passively, with the animal chewing and then apparently ‘retching’, followed by opening of the mouth and oral expulsion. This description is more like regurgitation and/or spitting, not actively/forcibly ejecting the contents (see [69], Supplementary Material Video S1). Therefore, this additional report of ‘vomiting’ appears to fit our conclusion that these instances of ‘vomiting’ in rodents have more in common with regurgitation followed by gagging rather than emesis *per se*, but this conclusion requires formal testing (see Section 5.4).

From studies on emetic species [12,14,16,30,50,117,118], forcible expulsion of gastric contents requires the following:

- Longitudinal shortening of the oesophagus;
- Intense contraction of the costal diaphragm with concomitant inhibition of contraction of the crural (circumoesophageal) diaphragm;
- Relaxation of the upper and lower oesophageal sphincters;
- Compression of the stomach by intense contraction of the rectus abdominis muscle.

We now examine these criteria, which can be used to define vomiting to further inform our conclusions regarding the ability of rodents to retch or vomit.

#### 5.3.1. The Characteristics of the Gastroesophageal Junction in Rodents Makes Vomiting Problematic

For gastric contents to enter the oesophagus, including during vomiting, they must pass through the ‘anti-reflux barrier’: a region of elevated pressure located between the stomach and the oesophagus. The main components are the circular smooth muscle of the lower oesophageal sphincter (LOS) at the gastroesophageal junction and the striated muscle of the crural diaphragm encircling the oesophagus. In humans, carnivores (e.g., ferret, cat, dog) and the insectivore *Suncus murinus* (see [3] for refs), the crural fibres encircle the LOS region, giving rise to a single zone of elevated pressure [119,120]. However, in the rat, there are two zones of elevated pressure because the diaphragmatic crus and the LOS are separated by a relatively long intra-abdominal segment of the oesophagus [121,122]. Thus, overcoming this powerful barrier against reflux to allow for vomiting to occur could be difficult.

Anatomical studies of species that are classically defined as vomiting and non-vomiting showed that the intra-abdominal segment of the oesophagus was relatively longer in non-vomiting species and in some species, it was narrower, resulting in a higher luminal resistance to flow [3]. The importance of the length of the intra-abdominal segment of the oesophagus is illustrated by two clinical observations: (i) children in which the intra-abdominal segment of oesophagus is abnormally short are particularly prone to GOR [123]; and (ii) a surgical procedure called Nissen fundoplication is used in specific patient groups to treat GOR and involves lengthening the intra-abdominal segment of the oesophagus. Modelling this operation in the ferret has shown that in response to an emetic, it increases the number of retches while markedly decreasing vomits [123]. This shows that lengthening the intra-abdominal oesophagus increases the resistance to the flow of vomitus, which may explain why rodents seem to retch without progressing to vomiting. In rodents, the strong anti-reflux barrier combined with a relatively long intra-abdominal oesophagus will impede the passage of contents in bulk to the oesophagus, leaving only retches. Whilst the relative length of the intra-abdominal segment of the oesophagus may differentiate species with an established ability to vomit from those which do not, there is no evidence that the longitudinal distribution of smooth and striated muscle in the oesophagus is related to vomiting. In humans, the proximal 1/3 is striated and the distal 2/3 is smooth muscle, but in cats, dogs, ferrets, pigs, rats, rabbits, guinea pigs, beavers, squirrels and ground squirrels, the striated muscle is considerably more extensive [3,119,124].

In contrast to species with a clearly established emetic reflex, rats and mice do not have natural GOR; hence, to use them as models for GOR usually requires some form of surgical intervention, often including modification of the abdominal oesophagus (see [125], Table 2). Chronic obstruction of the pylorus in rats results in high levels of gastric acid secretion, which accumulates and eventually refluxes as the stomach becomes over distended. This does not mimic ‘natural reflux’ but does illustrate that under extreme conditions, liquid may be able to pass from the stomach to the oesophagus in the rat. However, even in conditions when there is a marked degree of gastric stasis (e.g., induced by the chemotherapeutic agent cisplatin) leading to accumulation of solid gastric contents and marked chronic distension of the stomach, neither retching nor vomiting has been reported in mice or rats (e.g., [111,126,127]). Additionally, in cisplatin-treated rats, significant gas accumulation has been reported, indicating that rats may have a limited ability to belch (eructation) [127], which, like GOR and vomiting, also requires dissipation of the anti-reflux barrier. Belching also requires relaxation of the upper oesophageal sphincter (cricopharyngeus) [128]. Almost all human patients with retrograde cricopharyngeus dysfunction have an inability to belch, but as just over half also have difficulty vomiting [129], the reflex control of this muscle in rodents should be considered when trying to understand their inability to vomit forcefully.

The morphological relationship between the terminal oesophagus and the proximal stomach (angle of His) may also contribute to the anti-reflux barrier by forming a ‘flap valve’ [119]. In the rat there is a fold (‘limiting ridge’) of tissue between the forestomach and glandular epithelium, which is prominent at the point where the oesophagus ‘enters’ the stomach [130], forming mucosal folds obscuring the oesophageal lumen [121,131]. In humans and other emetic species, the distal oesophagus enters the stomach more laterally than in non-emetic species [3]. In addition to these anatomical features that limit GOR in rodents, in comparison to humans and carnivores, there are also functional differences:

(a) For GOR, eructation and emesis to occur, a dissociation of the contraction of the crural (encircling the oesophagus) and costal diaphragm is needed; both contract during breathing and retching, but during GOR or vomiting, the former does not, while the latter is contracting. During swallowing, the rat was unable to uncouple the contraction of the crural and costal regions [132], leading to the suggestion [133] that, unlike the cat, rats are unable to exert “*separate and selective*” control of them and that this may contribute to their inability to vomit. However, a subsequent study electrically stimulated the superior laryngeal nerve in the rat at high frequency (20–50 Hz), inducing ‘severe gagging’, and concurrent activation of the abdominal muscles and costal diaphragm were seen while the crural part was inactive [89]. It should be noted that the costal diaphragm activity was not greater than during normal breathing as might be expected during a gag, retch or vomit. Uncoupling of the costal and crural diaphragm in the rat also occurs in the post-inspiratory phase of eupnoea [134]. However, even if the rat can inhibit the crural fibres, passage of gastric contents into the oesophagus may still be problematic, as selective destruction of the crural sling alone is insufficient to allow GOR to occur [135].

(b) In emetic species, a longitudinal shortening of the oesophagus occurs during retching and vomiting, shortening the intra-abdominal segment of the oesophagus. Shortening, changes the morphological relationship of the entrance of the oesophagus to the stomach, and with the accompanying proximal gastric relaxation, the potential ‘flap valve’ created by the angle of His is thus abolished. These physiologically driven morphological changes will facilitate the passage of gastric contents into the oesophagus [10,117].

Oesophageal shortening is under vagal efferent control. In *Suncus murinus*, vagal efferent stimulation at a relatively high frequency shortened the oesophagus by 39 ± 6% of the resting length compared to only 5.0 ± 2% in the mouse [136]. This limited ability of rodents to shorten their oesophagus means that they have a reduced ability to eliminate the resistance to the flow of gastric contents, making ejection of vomitus challenging. Further, an elongated segment of intra-abdominal oesophagus would be more prone to ‘kinking’ if the rectus abdominus contracted, pushing the stomach towards the diaphragm, as occurs during vomiting.

(c) In addition to inhibition of the crural diaphragm, relaxation of the LOS itself by nitrergic and other inhibitory neurones is necessary to further reduce the resistance to the flow of material from the stomach to the oesophagus. The percentage of myenteric plexus nitrergic neurones in the LOS was noted [137] to be, in general, lower in species that are able to vomit (e.g., human, cat, monkey, opossum) compared to those considered to be unable to vomit (e.g., mouse, rat, guinea pig, horse (but see [110])). This difference in neurones indicates a higher baseline LOS tone in the non-vomiting species, as a greater number of inhibitory nitrergic neurons would be needed to relax a more contracted LOS. This interesting hypothesis is experimentally testable.

The key anatomical and functional differences in the oesophago-gastric region of rodents and species with a well-defined ability to retch and vomit are summarised in Figure 2.

#### 5.3.2. The Genesis of the Abdomino-Thoracic Pressure Gradient

The key propulsive force for the ejection of gastric contents is the force generated by contraction of the diaphragm and abdominal muscles. Limited anatomical studies [3] have overall shown a greater muscle density/unit area in emetic compared to non-emetic species, implying greater force generation. Caution should be exercised, as the diaphragm may also be adapted for other functions (e.g., diving, posture, locomotor–respiratory coupling) [138]. Also, anatomical measures take no account of the functional consequences of the differences in end-plate distribution seen between cat and dog and rat and rabbit diaphragms: a single continuous band in the latter and from two to four discontinuous bands in the former [139].

The force generated by the diaphragm under specific conditions (e.g., eupnoea, hypercapnia) is measured through transdiaphragmatic pressure (Pdi) and the maximal force (Pdimax) is achieved with bilateral phrenic stimulation or naturally through intense expulsive events (gag, cough, sneeze, vomit). These maximal expulsive events require rapid (<1 s) recruitment of all the motor unit types in the order S (slow contraction, fatigue-resistant) > R (fast, fatigue-resistant) > Fint (fatigue intermediate) and finally FF (highly fatigable) [140]. There is some evidence [141] for differences in the proportion of S+R and FInt + FF units between non-vomiting species (rat ~65% and hamster ~54%) and an emetic species (cat ~34%), but there is insufficient published comparative data to draw a firm conclusion. In the rat, an increase in the intragastric pressure of ~50 mmHg was recorded during a sneeze (i.e., approximating Pdimax) [140], which is comparable to that recorded in the cat and ferret during vomiting [14,19] and greater than in *Suncus murinus* during retching [95]. Mice also appear to be capable of generating comparable intra-abdominal pressure changes, as the PDimax is ~50 mmHg [142] and during a ‘cough-like’ response, an intra-thoracic pressure of ~60 mmHg was recorded [100].

Although additional data are required, the ability to generate a sufficient change in intra-abdominal pressure does not seem to be the primary reason for the inability of rodents to vomit semi-solids (or more forcibly, fluids). The reason is more likely to reside in the anatomy and physiology of the anti-reflux barrier (see above).

The above arguments do not exclude the possibility that small amounts of gastric contents could enter the terminal oesophagus and reach the proximal oesophagus by oesophageal contraction (known to occur during ‘severe gagging’ [89]), then being ejected by dropping from the mouth or gagging. However, rats have died of choking on regurgitant following ingestion of non-nutritive high-bulk diets [143], suggesting that pharyngeal clearance of semi-solids can be problematic. Liquids are more likely to reflux into the oesophagus than solids. In the rat, gastro-oesophageal reflux of a viscous solution containing a contrast medium was demonstrated radiologically, following i.v. administration of a NK_1_ receptor agonist with the duration being 2–6 min, depending on the dose [144]. Fluid in the oesophagus could enter the mouth, possibly aided by oesophageal contraction, but ‘regurgitation’ can readily be distinguished from vomiting [145].

#### 5.3.3. Conclusions on the Ability or Inability of Rodents to Retch and Vomit

Rodents, as exemplified by laboratory mice and rats, are unable to vomit in a way that is comparable to emetic species. However, under specific circumstances, they do appear to be able to regurgitate gastric contents and this may explain the rare reports of occurrences of ‘vomiting’. The ‘retching-like behaviour’ and ‘severe gagging’ has some similarities to responses seen in emetic species, but also differences. In rodents, a clear distinction between the rare ‘retching-like behaviour’ and the ‘severe gagging’ has not been demonstrated.

The presence of ‘retching-like’ (mouse and rat) and ‘severe gagging’ (rat) behaviour is demonstrated by observational and EMG studies, but the rarity or absence of vomiting that is comparable to that observed in emetic species suggests that the emetic reflex in rodents is degenerate or incomplete, rather than absent. This is consistent with the argument that rodents have ‘lost’ the ability to vomit during their evolution: a function found in progenitor mammalian species [110]. Rodents show evidence of changes in molecular structures that are otherwise involved in mechanisms which stimulate vomiting; in rodents, the motilin receptor is now represented by a pseudo-gene, and compared with humans, the number of 5-HT_3_ receptor channel subunits and the potency of some NK_1_ receptor ligands is markedly reduced (see [110] for discussion and references). The implications of this conclusion are discussed in Section 6, but first, we propose experimental approaches to resolve the discrepant views in the literature.

### 5.4. Experimental Approaches to Resolving the Rodent Vomiting Question

We propose the following as a starting point for studies to resolve this issue.

(a) Species selection. Initial studies should be undertaken ideally on mice and rats of the same strains, weight and sex as those in Table 1, Table 2 and Table 3. Although we did not identify any strain/regional trends in the reports of ‘emesis’ amongst rodents, the sample is not large. Comparing the presence of ‘emetic-like’ responses between different strains and species of rodents in a greater number of studies would be essential to fully address this question.

If rodents which have been laboratory bred for many generations have an incomplete or degenerate emetic reflex, then one might expect some of their wild equivalents to have retained this ability in view of the selection pressure in the wild vs. the laboratory environment. Studies of neophobia and pica have been undertaken in wild-caught rats and although these studies did not report vomiting [146,147], consideration should still be given to further investigating wild-caught rats and mice. If convincing data for emesis is obtained in any species or strain, then tissue samples (e.g., brainstem) should be preserved for anatomical and genomic analysis so that the genotype and phenotype of the animal can be defined for comparison with non-responders.

(b) Feeding status. Details of the feeding status (i.e., fed *ad libitum* or food deprived for a stated period) of the animal must be reported so it is clear whether any gavaged liquid is mixing with the food or is given on an ‘empty’ stomach. This will also increase confidence about identifying ejected material as gastric contents (i.e., vomit). The volume of any gavaged liquids should be kept within physiological limits, as high volumes are likely to promote reflux [94].

(c) Behaviour. As individual events of ‘retching-like behaviour’ can be brief, especially in mice, high resolution video recording is essential and this also permits assessment of mouth opening [35,36,37]. It is essential that an adequate time is allowed for any potential emetic agent to manifest its effects, resulting in several hours of recording for some substances (e.g., cytotoxic anti-cancer drugs, infectious agents). Classifying a behaviour as retching or vomiting based solely on observation will always be subject to some degree of uncertainty and when using pharmacological agents, additional behaviours (e.g., prostration, tremor, convulsions) may be evoked, which confounds observations (see descriptions in Table 1 and Table 2). Physiological measurements will eventually be needed to correlate the key parameters (see ‘*e*’ below) with behaviours to be certain that the observed event is either retching or vomiting. However, automated analytical techniques such as those developed for the recording and analysis of emetic responses in *Suncus murinus* [99,148,149] could be used as an initial basis for comparison with ‘emetic-like’ behaviours in rodents. Emetic agents should be tested in animals with purely liquid gastric contents, as well as in animals that are fed normally, to test the capacity for ejection, as liquids may be expelled more readily and/or forcibly than solids/semi-solids. Whilst direct observation will provide key evidence about the ability to vomit, if material is not ejected forcibly, the underlying reason will remain unknown, so additional physiological studies will be required.

(d) Stimuli and doses. The three main inputs (abdominal vagal afferents, area postrema and vestibular system) to the NTS that are capable of inducing emesis in diverse species are present in rodents (exemplified by rats and mice) and when stimulated, they can induce pica and/or aversive/avoidance responses [15,43,44,53]. Neurophysiological studies on rats have shown activation of AP neurones and vagal afferents by substances that are capable of inducing emesis in other species (e.g., Area Postrema: apomorphine [150]; adenosine triphosphate [151]; arginine vasopressin [152]; lithium chloride [150]; abdominal vagal afferent: cholecystokinin-8 [153]; cisplatin [154]; copper sulphate [155]).

However, activation of these emetic inputs to the NTS does not mean that retching or vomiting will occur, as this requires the pathways connecting the NTS to the motor outputs to be functional. The recent mouse studies [35,37] using optogenetic stimulation of the NTS indicate that the pathway from the NTS to the Amb/RVLM is functionally intact and can cause ‘retching-like behaviour’. However, optogenetic stimulation is an intense stimulus (c.f. focal electrical stimulation), so it should not be assumed to equate to physiological activation of the NTS via its anatomical inputs. In addition, the nucleus ambiguus region contains the vagal motor neurones innervating the striated muscle of the oesophagus [92,93] and is involved in the gag reflex pathway [86], so stimulation could be causing or facilitating regurgitation, oesophageal retro-peristalsis and gagging instead of ‘true’ emesis.

We noted above that protoveratrine-A was the stimulus most often reported to induce ‘retching-like behaviour’ and, occasionally, ‘vomiting’ in rodents (see Table 1 and Table 2). Studies on dogs [156] and cats [157] with other ester veratrum alkaloids (including veratrine) demonstrated that these substances are potent emetics (even in ruminants [158]). Although initial studies identified the nodose ganglion as a critical site where they act to induce emesis [73,157], subsequent investigations showed a more widespread action on vagal afferents [156]. This is not surprising, as the main action of veratrum alkaloids is on voltage-gated Na^+^ channels, making it unlikely that the nodose ganglia are the only sites for their emetic action. The action of veratrum alkaloids on excitable tissues also explains their more general toxic effects in humans, including muscle weakness and bradycardia [159] and the marked neurological and locomotor disturbances reported in some rodent studies, which were occasionally lethal (see Table 1). If PVA or other veratrum alkaloids are used to confirm the early studies (e.g., [33]), careful selection of doses will need to avoid general neurological and cardiovascular effects; otherwise, it may not be possible to interpret the results. A mouse study reported that only the ester veratrum alkaloid derivatives caused ‘retching movements’ [33]; thus, it may be possible to determine the molecular mechanisms as being responsible for this effect and then better characterise the response.

Additionally, selection of doses to investigate an ‘emetic response’ in rodents is important, as some emetics have a bell-shaped dose–response curve in emetic species (e.g., ferret: apomorphine [52]; morphine [160]). Further, there are marked species variations in sensitivity to a range of emetic stimuli (e.g., see [7], Table 1). As studies will be attempting to definitively identify if rodents are able to retch or vomit, it is essential that negative results are not false negatives because of dose selection.

(e) Physiological parameters. A definitive answer regarding ‘retching’ and ‘vomiting’ in rodents can only be obtained by measurement of key physiological parameters in either conscious or decerebrate animals, enabling comparison with similar studies on emetic species. Both gagging and retching have been evoked in emetic species under general anaesthesia, with urethane (e.g., ferret [67,107]) providing an alternative option. Such studies will also identify any factors constraining the ability of rodents to vomit in the same way as emetic species.

Retching and vomiting are essentially modifications of respiratory muscle activity and a range of methods have been developed for monitoring respiration in mice and rats (see [161] for review). The key measurements are recordings of the electrical activity of the costal and crural diaphragm, together with abdominal muscles (e.g., external oblique). During episodes of retching (or a single gag), all three muscles will be synchronously active, but during a vomit, the crural fibres should fall silent while the activity in the costal and abdominal muscles will be more prolonged and intense than during a retch.

Alternatively, simultaneous recordings of intra-thoracic (ITP) and intra-abdominal (IAP) pressure will be needed to allow a clear differentiation of retching from vomiting. During retching, ITP is reduced coincident with increased IAP, whereas during a vomit, both pressures increase with a greater magnitude and duration. If the studies are undertaken in conscious animals, parameters should be monitored using surgically implanted telemetry systems, as used in shrews and ferrets [99,162,163,164,165]. EMG recordings have the advantage of providing information on the status of the crural diaphragm, as although the IAP may increase, the distal oesophagus and gastro-oesophageal junction in rodents may limit the forcible ejection of gastric contents.

Ideally, EMG recordings should also be made from the digastric (jaw opening) muscle to improve the temporal correlation between parameters. Recordings from the thyrohyoid muscle (a laryngeal elevator) would also be helpful, as activity has been recorded during gagging in rats [85], allowing for comparison with recordings in dogs during retching and vomiting [118].

To summarise, the key physiological differences that would differentiate retching and vomiting from externally visible, apparently similar events (e.g., cough) in a rodent are as follows: differential activation of the crural and costal diaphragm; longitudinal shortening of the oesophagus; relaxation of both oesophageal sphincters; and the temporal relationship between the preceding parameters and abdominal and intercostal muscle activity.

## 6. Conclusions and Implications

### 6.1. Conclusions

- The reason why ‘retching-like behaviour’ has only been sporadically reported in rats and mice is unclear. Perhaps it may have been mistaken for other similar behaviours (e.g., sneeze, cough) or signs of neurotoxicity, especially as several reports used neuroactive veratrum alkaloids. It is also possible that ‘retching’ or ‘vomiting’ have been observed more frequently, but the finding has been dismissed as anomalous or has proven difficult to publish, as is known to occur with ‘negative data’ [166].
- As exemplified by rats and mice, rodents have a ‘retch-like response’, but the response is small, brief and not comparable in magnitude to that seen in emetic species and could easily be missed by an observer. The limited data on intragastric pressure during ‘retching-like behaviour’ in mice indicates a weak response that is not comparable with that seen in emetic species, despite data suggesting that the mouse diaphragm can generate similar forces.
- Behaviours described as ‘vomiting’ are very rarely reported and the descriptions are not convincing compared to the forceful ejection that occurs in established emetic species, especially when the constraints of the GOR barrier on the bulk flow of semi-solid material into the oesophagus are considered.
- Until additional physiological studies are undertaken to clarify the events underlying ‘retching-like behaviour’ and to better define ‘vomiting’ in mice and rats and other rodents, we suggest that sweeping statements such as “rodents do not vomit” are avoided and are qualified by more specific references to particular species, details of what was observed or measured and under which conditions. The terms ‘retching-like’, ‘pseudo-vomiting’ or ‘pseudo-emesis’ may be more appropriate descriptions until the exact physiological characteristics of the events are defined.
- These terms are consistent with the proposal that during evolution, rodents have ‘lost’ the ability to vomit yet retain some ancient traces of the emetic reflex (and its molecular constituents) that may be more or less apparent in different species, strains or in-bred/wild animals (see below).
- The preliminary evidence for the presence of retching and the possibility of regurgitation or vomiting under specific circumstances (e.g., liquid gastric contents) has a number of implications for biomedical science and rodent biology, as discussed below.

### 6.2. Implications

(a) Pharmacology and toxicology. Convincing evidence that either rats or mice have the ability to retch and vomit would have major implications for toxicology, as both events would be regarded as adverse effects of a potential drug [4,38,40]. The presence of emesis, even in specific species/strains of rodent, could facilitate earlier identification of emetic liability of a potential drug and, because of the molecular tools available in rodents, could help identify mechanisms to reduce/remove such liability.

Pica and conditioned taste/flavour aversion are well-established as metrics in rodents to identify that a novel agent has the potential to activate emetic inputs to the brainstem (Chapter 8, [15,36]), but it is argued that they may be more equivalent to nausea [9,15,53]. Taking this view, the presence or absence of pica and/or an aversive response in a rodent in response to a drug would not exclude the possibility for the induction of retching and vomiting by that drug in a responsive species. Therefore, formal testing of the induction of emesis in a known emetic species would still be needed to fully exclude emetic potential. However, a well-defined ‘emetic response’ in a rodent would be advantageous for improving the predictive validity.

Recording physiological data (e.g., diaphragm EMG, IAP, IGP) is impractical for routine toxicological screening, so high-resolution video recording will be required, with subsequent automated analysis. It may be possible to incorporate such analysis into the behavioural observations used: for example, the modified Irwin Screen [41] for CNS toxicity.

Additionally, rats, mice and other rodents (e.g., hamsters) are used widely for identification of the pathophysiologic effects of different toxins [39], with the data often being used to predict the effects that the exposure to the toxin will have on humans or other target species. The mechanism of the toxicity is commonly investigated first in rodents, and notably, the lack of an emetic response in rats and mice has led to the assumption that larger doses of a toxic compound can be administered orally without compromising evaluation due to emesis [39]. However, this use of a high dosage could cause the over-estimation of predicted human toxicity [39], and if some rodents are able to regurgitate/vomit the ingested contents, then their validity for predicting toxicity in emetic species (including humans) will increase. The latter would also require carefully monitoring for the occurrence of these responses, because any unexpected or non-observed oral expulsion of the administered toxin would compromise the study.

(b) Animal variability and reporting anomalies

It is essential that in any publications in this area, precise details are given about the animals following the ARRIVE guidelines [167]. Strain may be of critical importance, so full details should be reported as there is considerable genomic variation between inbred strains [168], and phenotypic differences have been reported between the strains of rats from different suppliers [169]. There is some evidence for strain differences in mice and rats in the pica response to emetics [111,112,147] and even in the emetic species *Suncus murinus*, there are both strain and sex differences in emetic sensitivity [113,114,170]. The age of the animal may also be relevant, as aged mice are reported to be less susceptible to motion sickness [171].

Although there may be an assumption that a behaviour observed over the course of the research using rats or mice is not retching or vomiting, our analysis suggests that such observations should not be dismissed. We suggest that unusual behaviour should be video recorded for detailed analysis and if this is not possible, at least reported against the background provided by this review. Our focus has been on laboratory-based studies, but filming animals in nature can fortuitously provide data on novel behaviours, including emesis (e.g., shark [172]; squirrel [69]).

(c) Rodents as biomedical models of retching and vomiting.

This reassessment raises the question of whether rodents can now be considered as models to investigate the mechanism by which toxins or drugs induce or prevent retching/vomiting. This is particularly relevant because of the tractability of genetic modification in mice (e.g., [35,37]). Currently, the data are insufficient to propose such use. High-speed video recording is required to reliably observe any ‘retching-like’ response (duration and mouth-opening angle), but of more relevance, the number of events seems small in comparison to the same stimuli in emetic species, and vomiting is either rare or does not occur. The anatomical arrangement of the anti-reflux barrier in rats and mice is also likely to be a confounding factor in interpreting data from rodents. Further, given the degeneration/changes in some molecular structures (e.g., motilin) involved in the stimulation of vomiting, relative to humans [110], there is still no guarantee that the ability of novel substances to stimulate these ‘emesis-like’ or ‘pseudo-emetic’ responses can be reliably translated to humans.

The reliability of retching/vomiting in rodents does not affect the utility of conditioned taste/flavour aversion paradigms as a model to study conditioned aversive responses involving activation of the area postrema, vestibular system or abdominal vagal afferents (see [9,53] for discussion), provided that the risk of not translating to humans is considered. Conditioned flavour aversion requires activation of the NTS, followed by subsequent activation of the lateral parabrachial nucleus, as shown by optogenetic stimulation [35], while the retching and vomiting require NTS activation of ventral brainstem pathways (see Introduction) to reconfigure the activation of the respiratory motor neurones and the DMVN/NA to initiate the accompanying digestive tract motility changes.

**Figure 3:**
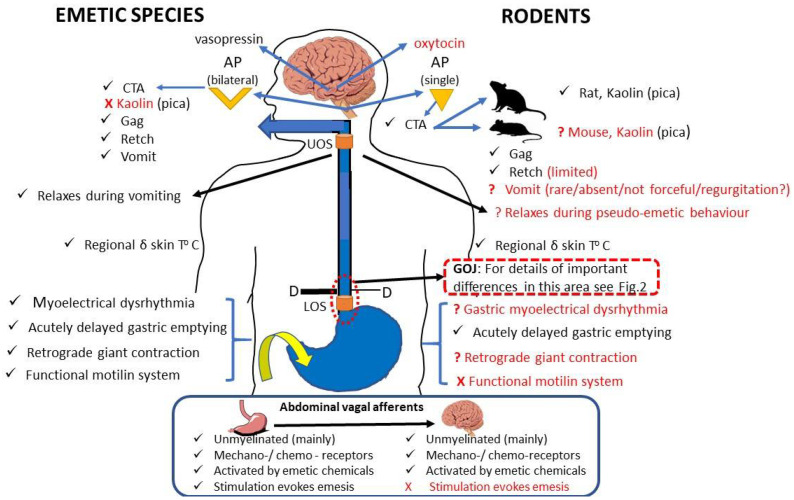
A diagram summarising major anatomical, physiological and behavioural differences between species with a well-defined emetic reflex (i.e., the ability to retch and vomit) and species without, or with evidence of a pseudo-emetic reflex, represented here by mice and rats. Major differences are identified with red text. For detailed discussion, caveats and additional references see text. The following list provides references to specific aspects identified on the figure: abdominal vagal afferent activation by emetic chemicals [153,154,155,173,174,175,176]; abdominal vagal afferent-evoked retching or vomiting [28,67]; acutely delayed gastric emptying [9,53,127]; area postrema (AP), [177,178], bilateral in all emetic species currently studied except for the shrew, *Sorex unguiculatus*, in which it is a central structure [96]; conditioned taste aversion (CTA) [9,15,53,179,180]; gastro-oesophageal junction (GOJ, see Figure 2); motilin [109,110]; gag [85,86,88,89]; kaolin consumption [15,53,111,146,181,182] but for discussion of pica in humans, which may be cultural in origin, see [15]; mechano-/chemo-receptor [173,174,183,184,185,186,187]; myoelectrical dysrhythmia [15,77]; oxytocin [15,188,189,190]; retch [10]; retrograde giant contraction [30]; skin temperature [51,54,57,191]; unmyelinated abdominal vagal afferents [183,184,185,186]; upper oesophageal sphincter (UOS) [128,129]; vasopressin [15,188,189,192]; and vomit [3,8,37,41].

In addition to the direct issues associated with retching and vomiting addressed here, there are also a number of other physiological differences between rodents and emetic species (summarised in Figure 3) which may also question the suitability of rodents as models with clinical relevance to humans.

(d) Rodenticides.

Toxins used to control rodent populations for public health reasons are used with the perceived inability of them to vomit being seen as advantageous in maximising the dose available for ingestion. Aversive responses are well-developed in rodents, so the induction of a learned avoidance to the food mixed with the toxin is a potential issue if the toxin is not lethal. Poisoned wild rats will ingest soil (geophagia) in an attempt to mitigate the effects of the toxin by adsorption or slowing gastric emptying with comparable ingestion of clay/soil observed in non-human primates and birds (see [15] chapter 8 for discussion and references). This mimics laboratory studies with kaolin ingestion following toxin administration, which has been shown to enhance recovery from illness induced by a toxic chemotherapeutic agent in rats [193]. If some strains or species of rodent have an ability to vomit/regurgitate reliably, then they will be at a selective survival advantage if the rodenticide elicits an emetic response and will progressively dominate the population.

This point can be illustrated by reference to the preparations used as rodenticides and extracted from the plant red squill (*Urginea maritima*) containing bufadienolide cardiotoxic glycosides, of which the main one is scilliroside [90,116]. It was used because of its safety and selectivity. Due to their inability to vomit, red squill ingested by rodents would fully exert its toxic effects, but if ingested accidentally by a species capable of emesis (e.g., dog, human), the strong emetic action would limit its toxicity [90,116,194,195,196]. However, if some rodents can expel ingested contents, the efficacy and selectivity of red squill as a rodenticide in this instance is diminished, particularly as there is an anecdotal indication that a rodent, the woodchuck (*M. monax*), may “*vomit from red squill*” [50]; similar reasoning could be applied to current rodenticides.

(e) Rodent evolution.

Rodentia represent over 40% of all mammalian species, with five suborders and >2000 species [5,6,110]. Whilst rats and mice are the most relevant species for laboratory research, only a fraction of rodent species have ever been formally tested for emetic sensitivity.

With the possible additional exception of Lagomorpha (e.g., rabbits, hares, pikas), comprising the cohort Glires with Rodentia, there is evidence for the ability to vomit in representatives of all other major mammalian classes [110]. Thus, an inability to vomit is the exception among mammals. This, together with the identification in rodents of pseudogenes for proteins of potential relevance to the emetic pathway, implies that ‘rodents’ have lost the ability to vomit, rather than never having possessed it (i.e., the ancestral rodent had an intact emetic reflex). The evolutionary pressure for this to occur is not known, although heightened toxin detection by taste and enhanced hepatic detoxification mechanisms have been proposed [1,9,53].

Rats and mice derive from the genera Rattus and Mus, respectively, and are part of the suborder Myomorpha ([197], Figure 3). Interestingly, there is evidence that Sciuromorpha diverged earlier than Myomorpha [198,199]: Sciuromorpha includes the grey squirrel (*S. carolinensis*), which was recently reported to have retching and regurgitation/vomiting [69], and the woodchuck (*M. monax*), which [87] reported to be possibly able to vomit from red squill. Therefore, it is possible that those rodents which diverged earlier (e.g., Sciuromorpha) retained more intact emetic-like capabilities from the common rodent ancestor than those which diverged later (e.g., rats and mice). However, Horn et al. [3] tested the mountain beaver (*A. rufa*), from the suborder Sciuromorpha ([197], Figure 3) but found no evidence of emesis, perhaps suggesting that retention of emetic-like capabilities is confined to certain families, reflecting adaptation to specific environments. Further wider testing and/or observation of other rodent representatives could offer insights into the potential relationship between the presence of emetic-like behaviours and their position on the evolutionary tree.

Additionally, there is evidence for endogenous mechanisms that are capable of suppressing emetic pathways (e.g., pulmonary vagal afferents [200]; modulation of brainstem emetic pathways by cannabinoid_1_, GABA_B_ and µ opioid receptor agonists, most likely in the NTS: for references see [8,9,201]). An involvement of these mechanisms in suppressing emesis in rodents is testable by using chemogenetic techniques. A study on mice [202] identified AP neurones expressing the glucose insulinotropic peptide receptor (GIPR), which, when activated by the gut hormone GIP, suppresses the activation of AP neurones expressing growth/differentiation factor-15 receptors (GFRAL), resulting in a reduced drive to brainstem pathways mediating conditioned flavour avoidance, which is argued to be an index of activation of emetic-related pathways. A population of inhibitory AP neurones also express the ghrelin receptor [203]. Combinations of post-prandial hormones (including GIP) are proposed to suppress the normal protective role of the AP to permit exploitation of “*calorie-rich foods containing small amounts of harmful chemicals*” [202]. This is of relevance to the emetic reflex, as in emetic species, both GIP and ghrelin can reduce retching and vomiting induced by the cytotoxic anti-cancer drug cisplatin [79,204], raising the possibility that emetic pathways in mice (and other rodents) are potentially modulated both in the NTS (above) and the AP.

**Figure 4:**
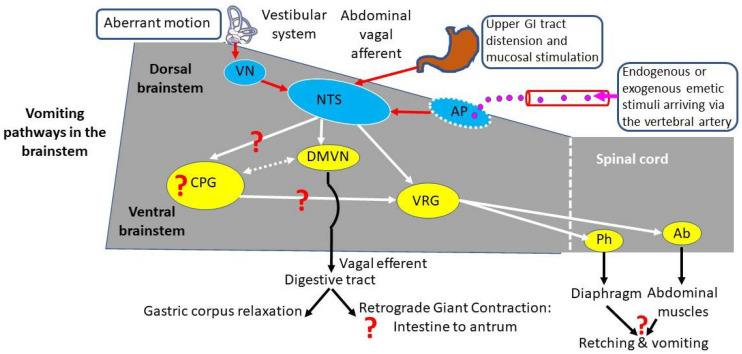
Diagram to illustrate the brainstem sites, where we hypothesise that there may be limited structural or functional connectivity in rodent brainstems (indicated by **?**), which may limit their ability to retch or vomit. There are data to show that the major inputs (vestibular system [51,56,191,206,207,208,209,210,211], area postrema (AP) [26,59,178], abdominal vagal afferents [153,154,155,173,174,175,176,183,184,185,186,187,206]) evoking emesis in species with a well-defined emetic response are present and active in rodents and connect with the nucleus tractus solitarius (NTS), which is considered to be a major integrative nucleus. Vagal efferents originating in the dorsal motor vagal nucleus (DMVN) supply the digestive tract and can produce gastric relaxation in rodents. However, the presence of a retrograde giant contraction [30] has not been demonstrated in rodents. Respiration is regulated (in part) by the outputs of the ventral respiratory group (VRG) of neurones to the phrenic neve nucleus (Ph) and abdominal (Ab) motor neurones in the spinal cord, and whilst rodents are capable of complex respiratory reflexes (e.g., cough [100,212,213]), it is not clear if there is an equivalent to the central pattern generator (CPG) for emesis, which, in emetic species, is responsible for coordinating the respiratory and abdominal muscles involved in retching and vomiting [23,24,25]. Alternatively, when the NTS receives an input which would result in an adequate stimulus to evoke emesis via the CPG, this output may not be sufficiently activated in rodents. For references and further details, see the text Figure modified from [8], which also has detailed references to brainstem emetic pathways.

Although rats and mice have functional ‘emetic’ inputs to the NTS, the drive from the NTS to the ventral brainstem central pattern generator (CPG; [8,17]) may be functionally degenerate, or the CPG itself may not be capable of the reconfiguration required for emesis, although it is still capable of generating the motor pattern for sneezing, coughing and gagging (Figure 4). The absence of a ‘vomiting centre’ or its existence in a rudimentary state as an explanation for the absence of vomiting in certain species was first proposed in 1891 [74]. The inability to vomit has been reported in adult members of a family with hereditary ataxia, including a failure to vomit in response to apomorphine or ingestion of hypertonic saline in one investigated member [205]. Molecular studies on rodents and specific human clinical populations should be able to identify the nature of any central lesion responsible for the inability to vomit. Whilst we focus on brainstem pathways to explain the degenerate nature of the emetic reflex, we speculate that as the gastroesophageal reflux barrier constrains the ability to vomit (Section 5.3: abdominal oesophagus length [3]; crural diaphragm position [135], LOS inhibitory innervation [137]), the evolutionary pressure to degenerate the emetic reflex may have originated in digestive tract adaptations to habitat change. At present, there is insufficient comparative data to weigh the relative contribution of brain stem neurophysiological factors (Figure 4) versus digestive tract anatomical and physiological factors (Figure 2 and Figure 3) to the presence of a pseudo-emetic reflex in rodents.

## Data Availability

No new data were created or analyzed in this study. Data sharing is not applicable to this article.

## Supplementary Materials

The following supporting information can be downloaded at: https://www.mdpi.com/article/10.3390/biology15010035/s1, Table S1. A summary of historic data on “retching-like” behaviour in mice; Table S2. A summary of data of ‘retching-like’ behaviour in rats, guinea pigs and rabbits; Table S3. Summary of data in Xie et al., 2022 [35], Huo et al., 2024 [37] and Ding et al., 2025 [36].

## Abbreviations

The following abbreviations are used in this manuscript:

α-NAA	alpha-naphtoxyacetic acid
Amb/NA	nucleus ambiguus
AP	area postrema
Calb1	calbidin-1 gene
CNO	clozapine N-oxide
CPG	central pattern generator
CTA	conditioned taste aversion
CuSO_4_	copper sulphate
DMNV	dorsal motor nucleus of the vagus nerve
DVC	dorsal vagal complex
ED	effective dose
EEC	enteroendocrine cell
EECs	enteroendocrine cells
EMG	electromyography
F_int_	fatigue intermediate muscle fibre
FF	highly fatigable muscle fibre
GDF-15	growth differentiation factor-15
GFRAL	glial cell line-derived neurotrophic factor family receptor alpha-like
GIPR	glucose insulinotropic peptide receptor
GLP-1	glucagon-like peptide 1
GOJ	gastro-oesophageal junction
GOR	gastro-oesophageal reflux
5-HT_3_ RA	5-hydroxytryptamine type _3_ receptor antagonist
IAP	intra-abdominal pressure
i.g.	intragastric
IGP	intragastric pressure
i.p.	intraperitoneal
ITP	intra-thoracic pressure
i.v.	intravenous
LOS	lower oesophageal sphincter
Na^+^	sodium ion
NK_1_ RA	neurokinin-1 receptor antagonist
NMDA	N-methyl-D-aspartate
NTS	nucleus tractus solitarius
PVA	protoveratrine-A
R	fast, fatigue-resistant muscle fibre
RVLM	rostral ventrolateral medulla
S	slow contraction, fatigue-resistant muscle fibre
s.c.	subcutaneous
SEA	*Staphylococcus aureus* enterotoxin A
SLN	superior laryngeal nerve
Tac1	tachykinin precursor-1 gene
