# Supplementary material for: Emesis in Rodents: Present or Absent? A Critical Review of the Evidence and Implications for the Use of Rodents in Biomedical Research
Source: Biology (Basel). 2025 Dec 25;15(1):35. doi: 10.3390/biology15010035 (PMC12784885; doi:10.3390/biology15010035)
Supplement: Supplementary file 1 [file biology-15-00035-s001.zip › biology-3941103-supplementary.pdf]

Table S1. A summary of historic data on "retching-like" behaviour in mice. Data is presented in chronological order. The tabulated descriptions of 'retching-like' behaviour are as defined in the original publications. See text for further details. Abbreviations: i.p.= intraperitoneal; M=male; NS= data not stated in the publication; R= number of "retching-like" events as defined by the authors; s.c. =subcutaneous. Reference numbers are those used in the reference list of the original paper.

Strain and sex	Supplier	Country	Stimulus/<br>dose/<br>route	Response	Latency	Number of R-like or V events<br>N=respond /tested	Profile	Ref
NS; Adult M	NS	USA	Veratrum ester alkaloids; ED_{50} µg/kg; s.c.; Protoveratrine A 430; Protoveratrine B 560; Germitetrine 680; Germitrine 230; Neogermitrine 400; Germerine 110; Veratridine 450; Cevadine 500.	*"movement of head and front part of the body as in the vomiting act"* -described as "retching movement"	NS	**R**=NS<br>**N**= 5-10 /dose (usually 8)	NS	33
CF1; Adult, M	NS	Japan	Protoveratrine-A; ED_{50} 39µg/kg; s.c.<br>ED_{99} (in 25week old animals) 95µg/kg, s.c.	No description other than *"retching"*	10-15 mins after an initial few minutes in which there was *"motor hypotonia"*	Respond/tested = Almost 100% in 95µg/kg group	Most frequent at 20-25 mins reducing in frequency until ~40min.	32
ddY albino; M;	Kyudo Animal Laboratory	Japan	Protoveratrine-A; i.p.; 100µg/kg.	*"downward and quick opening of the mouth, with upward and downward movement of the thoracic skin"; described as* "retching" accompanied by salivation, preening and	NS	**R**=11.6±3.8 in 60 min.<br>**N**=10/10)	Peak at 20-30 min	31

				decrease in exploratory behavior				
ddY albino; M;	Kyudo Animal Laboratory	Japan	α- naphthoxyacetic acid; i.p.; 550mg/kg.	"*recurrent episodes of wide opening of the mouth*"; Described as "retching" accompanied by salivation, lacrimation and sometimes a sudden jump	5 min	**R**=15.8± 4.2 in 30 min **N**=10/10	Peak at 10-15 min	31
ICR; M	Institute of Cancer Research (ICR)	Japan	Aconitine; p.o.; 1 mg/kg per day	"Vomiting-like action" with mouth opening	3-15 min	**NS**	Lasted for~3h after dosing; response continued to appear and persist for hours even on the 22nd day of dosing	73
ddY	SLC	Japan	Protoveratrine -A; s.c.; 50-200μg/kg;	'Retching-like behaviour', mouth opening but also a separate 'hiccup-like' behaviour	10-15min	**R**=14.3 (mean) at 50μg/kg; 35.8 μg(mean) at 100 μg/kg; 34.9 at 200	Peak at 15 -20min.	74

						µg/kg (N=4) in 60min		

Table S2. A summary of data of 'retching -like' behaviour in rats, guinea pigs and rabbits. Tabulated descriptions of 'retching-like' behaviour are as defined in the original publication. See text for further details. Abbreviations: CTA= Conditioned Taste Aversion; F= female; i.g. = Intragastric; i.p.= intraperitoneal; i.v.=Intravenous; M=male; NR= not relevant; NS= data not stated in the publication; R= number of "retching-like" events; s.c. =subcutaneous; *= description from a translation of original paper. Reference numbers are those used in the reference list of the original paper.

Strain and sex	Supplier	Country	Stimulus/dose/ route	Response	Latency	Number of R-like or V events. N= respond /tested	Duration	Ref
**RAT**								
White; F pregnant	NS	USA	Horizontal rotation	*"Frequent and severe retching movements accompanied by defecation and micturition"*	NS	NS	NS	71
NS	NS	USA	NS; *"poisoned"*	*"The abdomen retches, the back arches, the head lowers, the mouth gapes, and the tongue protrudes"; described as "Behavioral indices of emesis"*	NS	NS	NS	72
TEX: (SD) AM; M	Timco Breeding Labs	USA	Nicotine; 0.5-3.0 mg/kg; i.p.	*"occasional retching"* in all dose groups	NS	NS	60-90 min for *"all observable symptoms"*	105

SD: M	SLC	Japan	Protoveratrine-A; 50-200μg/kg; s.c.	'Retching', tongue protrusion, mouth opening downward, forelimb straddling.	10-15min	Mean 16.6-36.8 (dose related)(N=4)in 60 min	30-60min (dose related)	74
**Guinea-pig**								
NS;<br>M and F	NS	USA	Protoveratrine (60% A and 40% B); 150μg/kg; i.p.	*"violent retching movements were noticed, which were followed by vomiting"*……….. *"The hind legs became immobilized in flaccid paralysis while vomiting continued"*	A few minutes	NS	NS	49
NS	NS	China	Copper sulphate; i.g.;	"*discomfort symptoms similar to vomiting*". Gastrointestinal tract electrical activity was recorded with the authors reporting "disturbances" increased frequency and amplitude) coinciding with the "symptoms similar to vomiting" *	3.7 min	NS	NS	83
**Rabbit**								
NS	NS	Japan	Protoveratrine -A; 10μg/kg; i.v.	*Retching act: "movement of the head and front part of the body as in the vomiting act……. Masticating movements of mouth, licking the lips, marked salivation, dyspnoea and cyanosis were always associated with the retching period"*	1-2 min	**R**=5 in 3-15min<br>**N**=17/18	3-15min	85
NS	NS	NS	Apomorphine	No description; anecdotal report of 'retching'	NS	NS	NS	185
**Grey squirrel**								
NS; F	In the wild	USA	'Spontaneous'-stimulus not identified	Retching defined as "attempting to vomit without bringing anything up" [182]	NR	9R, expulsion of viscous white liquid;<br>2R+5R, expulsion (brown);<br>4R, expulsion (brown)	Total obs period 1.36 min. Retches ~1/sec	86

Table S3. Summary of data in Xie et al.2022 [35], Huo et al., 2024 [37] and Ding et al., 2025 [36]. *Only data with the highest dose of SEA is shown in this table and the authors also studied other Staphylococcal enterotoxins (see text).** Also studied at other doses, stimulus parameters (optogenetic laser stimulation only) or route. Response shown here is the one of greatest magnitude with that stimulus.+ The data on 'duration' is derived from the histograms in the figures and is the "*total time spent in retching like behavior in each mouse calculated by adding the time spent in each mouth-opening action*" ([35] e4). Data on the duration of each mouth opening action is given in [35], Figure S1 and in Table S1, but is labelled in the Table as "Retching duration". ++ Only selected data on the DVC tachykininergic neurone activation is shown in this table. In Ding et al. [36] data estimated from Figures. For further details see text. Abbreviations: Amb= Nucleus ambiguus; CNO= Clozapine N-oxide; $CuS0_4$= Copper sulphate; EMG= Electromyography recording from either the diaphragm or abdominal muscles; F= female; i.g.=Intragastric /gavage; IGP= Intra-gastric pressure; i.p.=Intraperitoneal; M= male; N=Number of animals; NS= Not stated; NTS= Nucleus tractus solitarius; PVA=Protoveratrine A; R=retches; RVLM= Rostroventrolateral medulla; SEA=Staphylococcal enterotoxin A; V=Vomits. Reference numbers are those used in the reference list of the original paper.

Strain and sex	Supplier	Country	Stimulus/ dose/ route	Response	Latency	Number of R-like or V events. N= number of animals	Duration +
**Xie et al., 2022++ [35]**							

Wild-type; M.	Jackson Laboratory<br><br>Gem Pharmatech (gene modified)	China	*SEA; 0.3mg/kg; i.p.	*'Retching-like behavior':* wide mouth opening (video camera) and when measured associated with synchronous bursts of EMG activity from the diaphragm and abdominal external oblique muscles	87 ± 14 min	R=5±3 in 180min; N=9	~6sec
As above	As above	As above	**PV-A; 0.4mg/kg; i.p.	'Retching-like behavior' by video camera	NS	R=NS N=9	~6 sec.
As above	As above	As above	**$CuSO_4$; 120mg/kg; i.g.	'Retching-like behavior' by video camera	NS	R=NS N=9	~1 sec.
As above	As above	As above	**Doxorubicin; 10mg/kg; i.p.	'Retching-like behavior' by video camera	NS	R=NS N=7	~6 sec.
As above	As above	As above	**Cisplatin; 10mg/kg; i.p.	'Retching-like behavior' by video camera	NS	R=NS N=7	~5 sec.
Tac1-IRES-Cre	As above	As above	**Optogenetic activation of Tac1+ DVC neurons; 10mW, 20Hz	'Retching-like behavior' by video camera	NS but 'Immediate' from records	R=NS N=9	~6 sec.
Tac1-IRES-Cre	As above	As above	**Chemogenetic activation of $Tac1^+$ DVC neurons by SEA; Control (mCherry+CNO 1 mg/kg+SEA); i.p.	'Retching-like behavior' by video camera	88±13min	R=NS N=9	~10 sec.
Tac1-IRES-Cre	As above	As above	**Chemogenetic activation of $Tac1^+$	'Retching-like behavior' by video camera	15±2min	R=NS N=9	~20sec

			DVC neurons; (hM3Dq-mCherry+CNO 1 mg/kg)				
**Huo et al., 2024 [37]**							
Wild-type, genetically modified and with acute gastritis induced by 2% aspirin + 0.6 M HCl, 10 µL/g, i.g.; M&F	Jackson Laboratory	China	***B. cereus*; 10µL/g; i.g.; hourly for 3h.	*'Retching-like behavior':* wide mouth-opening angle (video camera) and where measured a simultaneous transient increase in IGP ± diaphragm EMG	~60min	R=5-29 in 3h<br>N=10	NS
vGlut2-ires-Cre	As above	As above	**Optogenetic activation of glutamatergic NTS neurons; 20mW; 5sec	*'Retching-like behavior':* wide mouth-opening angle (video camera)	NS but 'Immediate' from records	R=~4 in 5sec.<br>N=4	NS
Calb1^{+}	As above	As above	**Optogenetic activation of Calb1^{+} NTS neurons.; 20mW, 20Hz, 5 sec.	*'Retching-like behavior':* wide mouth-opening angle (video camera). Also, 10Hz stimulation induced 2 mouth-opening events and rise in IGP (<0.2mmHg) in 5 sec.	NS but 'Immediate' from records	R=~4 in 5 sec.<br>N=4	NS
Calb1^{+}	As above	As above	**Optogenetic stimulation of NTS-Amb-RVLM pathway; 20mW,20Hz	'retching-like behaviour' (video-recording). In a group of 4 animals, stimulation of the same pathway at 10Hz for 1 sec evoked a single rise in IGP (<0.2mmHg) coincident with wide mouth gaping and increased dipaphragm EMG activity.	NS but 'Immediate' from records	R= ~4 in 5 sec.<br>N=18	NS

FosCreEr, hM3D	As above	As above	**Chemogenetic stimulation of *B. cereus* TRAPed NTS neurons; CNO; i.p.	*'Retching-like behavior':* wide mouth-opening angle (video camera) and where measured a simultaneous transient increase in IGP	NS	R=~40 in 60 min.<br>N=5	NS
Calb1+, ChR2 group	As above	As above	Optogenetic stimulation of NTS-Amb-RVLM pathway; 20mW,20Hz	Repeated activation evoked *forceful vomiting"* - oral ejection of gavaged dragon fruit juice	NS	V= Occurred and vomitus weighed<br>N=6	NS
**Ding et al., 2025 [36]**							
Wild type	Gem Pharmatech	China	Exendin-4; 1-1000µg/kg; i.p.	"Retching-like behavior": *unusual wide-mouth-opening movements*	~80 min (1, 10 and 100µg/kg); ~50 min (1000µg/kg)	1µg/kg:<br>R=~4 in 3 h (~3-5)<br>N=10<br>10µg/kg:<br>R= ~6 in 3h (~5-7)<br>N=10<br>100µg/kg:<br>R= ~6 in 3h (~4-8)<br>N=10<br>1000µg/kg:<br>R= ~7 in 3h (~6-8)<br>N=10	NS
